# Facilitating maintenance of stormwater ponds: comparison of analytical methods for determination of metal pollution

**DOI:** 10.1007/s11356-022-20694-0

**Published:** 2022-06-01

**Authors:** Snežana Gavrić, Kelsey Flanagan, Heléne Österlund, Godecke-Tobias Blecken, Maria Viklander

**Affiliations:** Urban Water Engineering, Department of Civil, Environmental and Natural Resources Engineering, Luleå University of Technology, 971 87, Luleå, Sweden

**Keywords:** Solids, Metal bioavailability, Metal fractionation, Sediment quality assessment, Urban runoff treatment, Stormwater management, Environmental risk assessment, Nature-based solutions

## Abstract

Stormwater ponds are widely used for controlling runoff quality through the sedimentation of particles and associated pollutants. Their maintenance requires regular removal and disposal of accumulated material. This necessitates an assessment of material hazardousness, including potential hazard due to its contamination by metals. Here we analyze 32 stormwater pond sediment samples from 17 facilities using several chemical analysis methods (total extraction, sequential extraction, diffusive gradients in thin-films DGT, and pore water extraction) in order to consider the complementarity and comparability of the different approaches. No clear relationship was found between analyses that have the potential to measure similar metal fractions (DGT and either fraction 1 of the sequential extraction (adsorbed and exchangeable metals and carbonates) or pore water concentrations). Loss on ignition (LOI) had a significant positive correlation with an indicator of the environmental risk developed in this paper (∑ranks) that incorporates different metals, speciations, and environmental endpoints. Large variations in metal levels were observed between ponds. As clustering was limited between the different analyses, a comprehensive analysis of different parameters is still needed to fully understand metal speciation and bioavailability.

**Supplementary Information:**

The online version contains supplementary material available at 10.1007/s11356-022-20694-0.

## Introduction

Urban stormwater and snowmelt runoff convey a variety of substances (e.g., solids, metals, organic contaminants, nutrients) that may deteriorate the quality of water and sediments in receiving water bodies (Marsalek et al. 1997; Blecken et al. 2012; Becouze-Lareure et al. 2019; Brudler et al. 2019). To mitigate this negative impact, stormwater can be treated prior to discharge using stormwater control measures (SCMs). Among the most widely implemented SCMs are stormwater ponds (Starzec et al. 2005; Winston et al. 2013; Drake and Guo 2008), which remove solids and associated pollutants from stormwater through sedimentation. The accumulation of stormwater sediments in ponds has been reported to range from 0 to 10 cm/year (Yousef et al. 1990; Van Buren et al. 1996; Marsalek and Marsalek 1997; Färm 2002), over time resulting in large volumes of polluted sediments that can pose a risk to a pond’s treatment function (reduced storage volume for sedimentation, risk of re-suspension, etc.; Blecken et al. 2017) and its ecosystem (Søberg et al. 2016; Minelgaite et al. 2020). Thus, appropriate design and regular maintenance, i.e., sediment cleanout and safe disposal are necessary to sustain a pond’s long-term function (Al-Rubaei et al. 2017; Blecken et al. 2017).

In practice, planning for sediment disposal often involves only the consideration of total metal concentrations, mainly because it is easier and less expensive compared to more detailed approaches involving analysis of different chemical forms (metal speciation), their availability (including bioavailability) and mobility between the solid and dissolved phases. Total metal concentrations are important for comparison with regulatory guidelines and assessing the degree of sediment pollution; however, high total metal concentrations pose a risk only if metal burdens are or can become mobile/bioavailable (Marsalek et al. 2006). If potentially available fractions of particulate metals are dominant, metals may be released to the water phase during de-watering (Karlsson et al. 2016), or can be mobilized if affected by the changes in the water ionic composition, pH, and reduction/oxidation conditions (Marsalek and Marsalek 1997).

Sequential extraction methods (Tessier et al. 1979) are sometimes used to investigate metal speciation in pond sediments (Mayer et al. 2008; Karlsson et al. 2016). These methods use reagents of increasing strength in successive steps to quantify metal fractions of differing availability, i.e., from readily available fractions to those that are non-labile and little available (Martin et al. 1987). Such analyses are relatively expensive and therefore not commonly used in practice by stormwater managers.

One alternative to sequential extraction methods is the use of diffusive gradients in thin-films (DGT). DGT is an in situ method that measures metal chemical speciation developed for water, soil, and sediment (Zhang and Davison 2015). The technique has been applied to monitor labile metal concentrations and, thus, to provide an estimate of metal bioavailability in natural freshwaters (Meylan et al. 2004; Sigg et al. 2006; Uher et al. 2018), contaminated soils (Manzano et al. 2019; Xu et al. 2019), sediments (Xie et al. 2021), and in three studies of stormwater runoff (Dunn et al. 2007; Hayman et al. 2019; McDonald et al. 2022). Another analysis considered to provide a better indication of potential bioavailability (compared to total metal concentrations), is the analysis of the pore water of contaminated sediments (Hin et al. 2010). Compared to pore water extraction and analysis, the DGT method is easier and more time efficient (Degryse et al. 2003) and when deployed in situ, DGT can reduce the risks of speciation change during transportation, storage, and sample analysis (Han et al. 2019).

Finally, toxicity testing may be used to assess the bioavailability of adverse levels of metals (Burton Jr. 2010) and has been applied to stormwater pond sediments (Karouna-Renier and Sparling 1997; Karlsson et al. 2010; Tixier et al. 2012), pore water (Mayer et al. 2008), and overlying water (Karouna-Renier and Sparling 1997; Karlsson et al. 2010).

In this study, sediments from 16 ponds and one sub-surface sediment facility were sampled and analyzed with different methods to evaluate and discuss the comparability and complementarity of these methods and the suitability of each method to facilitate proper maintenance of stormwater ponds. The selected methods include the following: (i) total metal analysis, (ii) pore water concentration, (iii) chemical method for investigating metal mobility (five-step sequential extraction analysis), (iv) passive sampling using DGT that provides information on the free and easily dissociated metal concentrations, and (v) toxicity.

## Methods

### Description of study sites

Sediments from 17 SCMs (16 stormwater ponds and 1 subsurface sedimentation basin) were collected. The facilities are located in four Swedish municipalities (6 facilities each in Örebro and Stockholm, labelled Or1-Or6 and S1-S6, respectively; 1 pond in Östersund, Os1; and 4 ponds in Växjö, V1-V4). An “I” or “O” added to the label (e.g., Or1-I and Or1-O) indicates if the specific sample was collected close to the inlet (“I”) or outlet (“O”) of the pond. The ponds were constructed between 1988 and 2010 and located within mainly industrial and/or commercial catchments (nine facilities), mainly residential catchments (five facilities) and next to roads and highways (three facilities). More information about the facility characteristics is presented in Flanagan et al. (2021).

### Sediment sampling

Sediment samples were collected during October–December 2019 using a Kajak sediment core sampler (KC Denmark) lined with a stainless-steel tube and equipped with a 2-m shaft. Prior to each sampling, equipment was rinsed three times in water from the facility. Generally, ponds were sampled at the inlet and the outlet except in two facilities (S4 and V2) where outlet sediments were too loose to be collected. This resulted in 32 composite sediment samples (17 inlet and 15 outlet samples). Entire cores were mixed in a stainless-steel tray (~ 3 L) and representative samples were obtained by quartering. In cases where quartering was not possible due to sediments being too liquid, representative samples were spooned into jars. Samples were placed in coolers with ice packs during transportation and upon arrival were stored in the dark cold (4 °C) room until the analysis. Equipment blanks were carried out by submerging the sampling equipment in 1.2 L of purified water for the contact time corresponding to the longest that occurred in the field (5 h). The leached metal mass in 1.2 L blank sample (µg) was assumed to be spread throughout the 3 L of the sampled wet sediments (1.2 kg/L assumed sediment balk density), resulting in 0.003–0.11% of the total concentrations measured.

### Analytical procedures

#### Metal analysis

Six metals (Cd, Cr, Cu, Ni, Pb, and Zn) were selected for the study and analyzed by an accredited commercial laboratory (ALS Scandinavia AB, Luleå) using the methods described in Table 1. Limits of quantification (LOQ) for each metal analysis are presented in Table S1 in the Supplementary material.

**Table 1:** Description of different metal analyses (total, pore water, DGT, and five fractions of sequential extraction procedure (SEP))

Method/fraction	Size	Lability/inclusion/speciation
Total	< 2 mm	Total leachable and digestible fraction in 7 M HNO3 (heat assisted)
Pore water	< 0.45 µm	All < 0.45 µm truly dissolved and colloidal metals (free metal ions, metals bound to inorganic ligands, organic ligands, and mineral colloids)
DGT	< 5 nm	Truly dissolved and weakly bound to organic and inorganic ligands, exchangeable metals from the solid phase “Colloids (nanoparticles) other than complexes with humic substances are unlikely to be measured by DGT.” (Zhang and Davison 2015)
SEP, Fraction 1	Whole sample (ground prior)	Adsorbed and exchangeable metals and carbonates e.g., metals bound through electrostatic attraction on exchange sites on the surface and interface of negatively charged complexes of soils (Hall et al. 1996a)
SEP, Fraction 2	Whole sample (solid residue from Fraction 1)	Metals bound to labile organic forms (which are the forms associated with reaction sites such as those present in humic and fulvic substances; Hall et al. 1996b)
SEP, Fraction 3	Whole sample (solid residue from Fraction 2)	Metals bound to amorphous Fe/Mn oxides e.g., oxides existing as cement between particles or as a coating on particles (Tessier et al. 1979; Hall et al. 1996b)
SEP, Fraction 4	Whole sample (solid residue from Fraction 3)	Metals bound to crystalline Fe oxides
SEP, Fraction 5	Whole sample (solid residue from Fraction 4)	Metals bound to stable organic forms and sulfides

These metals are commonly associated with urban runoff and reported in previous research about urban stormwater sediments. They have also been identified as priority pollutants subject to regulatory action (Eriksson et al 2007). The general dominance of these six metals has been described previously (see e.g. the review by Huber et al. 2016).

##### Total metal concentrations

Total metal concentrations were analyzed on dried and sieved (< 2 mm) samples after leaching with 7 M HNO_3_, using Inductively Coupled Plasma Atomic Emission Spectroscopy (ICP-AES), following ISO 11885:2007 and EPA-method 200.7:1994. Subscript “T” is used to represent total metal concentration.

##### Pore water metal concentrations

Pore water was extracted without prior digestion by centrifuging wet sediment and filtering the supernate through a 0.45-µm filter; the filtrate was analyzed without prior digestion. The samples were then acidified with 1 ml of nitric acid (Suprapur) per 100 ml. Metal concentrations were determined using ICP-AES following SS EN ISO 11885:2007 and EPA-method 200.7:1994 and using Inductively Coupled Plasma Sector Field Mass Spectrometry (ICP-SFMS) following SS EN ISO 17294–2:2016 and EPA-method 200.8:1994. Subscript “PW” is used to represent dissolved metal concentrations in pore water.

##### DGT labile concentrations

Within 24 h after collection, DGT devices (standard DGT holder for soils with 0.8 mm APA diffusive gel, polyethersulfone filter membrane, and Chelex binding layer) were deployed in wet sediment samples stored in well-filled 100 mL plastic jars and exposed to the sediment for 72 h. During this time the jars were kept in an isothermal bag with ice packs. The temperature was logged inside the bag for samples from Växjö; as the rolling 72-h average varied little during this campaign, the average temperature (2.5 °C) was used for the other samples. After the exposure, the DGT devices were rinsed with Milli-Q water (Millipore). They were sent to an external laboratory where resin gels were eluted in 10 mL of 1.4 M HNO3 (Suprapur) for at least 24 h on a shaking apparatus, prior to further dilution and analysis. The recovery rate of the elution was 100% (Österlund et al. 2012). A single DGT device is deployed per sample. Previous research estimated the precision/repeatability of the DGT measurement as relative standard deviation from several duplicates to be 16% and 9% for Cu and Ni, respectively (Österlund et al. 2012), and less than 5.6% for As, Cd, Cu, and P (Kreuzeder et al. 2015). Metal concentrations in DGT elutes were determined using ICP-SFMS according to SS EN ISO 17294–1, 2 (modified) and US EPA Method 200.8 (modified). The measured concentration in the eluate was used to calculate the accumulated metal mass (*M*) which is used for estimation of the DGT labile concentration (*C*_*DGT*_) according to Eq. 1.

1 $$\begin{array}{c}C_{DGT}=\frac{M\;\Delta_g}{D_{mdl}\;A\;t}\end{array}$$

where $\Delta$ *g* is the total thickness of the materials in the diffusion layer (diffusive gel and filter membrane), *D*_*mdl*_ (cm^2^ s^−1^) is the diffusion coefficient of metal for the deployment temperature, *t* is the deployment time, and *A* is the sampling area.

Subscript “*DGT*” is used to represent DGT metal concentration.

##### Sequential extraction

The sediments were subject to a five-step sequential extraction analysis following the method adapted from Hall et al. (1996a, 1996b). The five successive fractions, which exhibit decreasing mobility, are shown in Table 1. The method for obtaining the extracts is presented in Table S2 in the Supplementary material. The total metal concentrations in the extracts after each step were analyzed using ICP-AES following SS EN ISO 11885:2007 (modified) and EPA-method 200.7 (modified) and using ICP-SFMS according to SS EN ISO 17294–2:2016 (modified) and EPA-method 200.8:1994 (modified). Samples were ground prior to the first extraction step. Subscript “Frac1” is used to represent metal concentration after first extraction step (Fraction 1), and similarly after other fractions (Frac2, Frac3, Frac4, Frac5).

#### Microtox acute toxicity test on solid samples

Toxicity was measured using the Microtox test, in which the inhibition of the luminescence emitted by the marine bacterium Vibrio fischeri NRRL B-11177 was determined after 15 min according to ISO standard using freeze-dried bacteria (CSN EN ISO 11348–3:2007).

#### General parameters

To better understand the metal analysis and the effects of sediment characteristics on the comparability of methods, ten general parameters were measured: pH, electrical conductivity (cond) [μS/cm], dissolved oxygen (DO) [mg/L], dissolved organic carbon (DOC) [mg/L], chloride (Cl) [mg/L], total organic carbon (TOC) [% DW], loss on ignition (LOI) [% DW], total Kjeldahl nitrogen (N) [mg/kg DW], clay and silt fraction < 2000 µm (%ClaySilt) and fraction of sand < 63 µm) (%Sand).

Determination of conductivity and DO was carried out using conductivity meter (WTW 3110 or WTW Multi 3630) and DO probe (YSI model 58 or WTW Multi 3630) by immersing the probes in the sediments. pH was determined after leaching, according to EN 12,176:^1998^. Cl was analyzed by liquid chromatography of ions according to CSN ISO 10304–1:2007 and CSN EN 16,192. DOC was analyzed according to CSN EN 1484:1997 and CSN EN 16,192. TOC was calculated from total carbon content according to CSN ISO 10694:1995 and CSN EN 13,137. LOI was analyzed according to EN 15,169:2007. Total Kjeldahl nitrogen was determined according to EN 16,169:2012. Particle size distribution was determined using a laser diffraction particle size analyzer Horiba LA-960. Before analysis, particles > 2 mm were removed by sieving; this fraction was also weighed.

### Data analysis

Out of 1536 observations (total, pore water, and DGT labile concentrations, as well as concentrations in five fractions of sequential extraction analysis), 5.3% of the values were non-detects (i.e., left-censored, see Table S3 Supplementary material).

For censored data, correlations were tested using the nonparametric Kendall’s tau test in the Nondetects and Data Analysis for Environmental Data package (NADA) in R. For noncensored data, the Spearman rho correlation test was computed in R. The correlations were considered significant if the *p* value is ≤ 0.01. Significant differences between the two groups of samples were tested using Peto & Peto generalized Wilcoxon test in NADA. This test was, for example, used when testing significant differences in metal concentrations between the group of samples with the toxicity reported and the rest of the samples.

In order to consider the partitioning of metals in each sample, the partition coefficient (K_d_, the ratio of total solid concentration to dissolved pore water concentration) was evaluated. It was only calculated when at least one of the two concentrations was quantified; when one of the concentrations was not quantified, it was set equal to LOQ.

In addition, a principal component analysis (PCA) was applied to compress and visualize the dataset and investigate the general correlations among different parameters (i.e., parameters that are grouped together positively correlate while parameters situated on the opposite side of the origin are negatively correlated). The PCA parameters included different metal concentrations (total, pore water, DGT, and Fraction 1 of the sequential extraction), and 11 general parameters (pH, cond, DO, DOC, Cl, TOC, LOI, N, C/N (ratio of TOC and N), %ClaySilt and %Sand). The software package SIMCA 17 was used to create score plots (showing the 32 pond sediment samples) and loading plots (showing the 35 parameters). Each variable was pre-treated using “mean centering” and “unit variance scaling,” which are default options in SIMCA (Eriksson et al. 2013). The data autofit was used to check the significance of each component based on the cross-validation method (Eriksson et al. 2013). For the purposes of the PCA, censored values were replaced with ½ LOQ, after confirming that three different methods for treating values < LOQ (replacement with ½ LOQ, leaving the values out as missing values and replacement with 0) have no effect on the conclusions from loading and scoring plots of PCA.

### Ranking table

The 32 sediment samples were ranked and compared to estimate their overall environmental risk (considering solid and water phase metal concentrations and toxicity). As no regulations apply specifically to stormwater pond sediments, in order to have a more robust conclusion of the risk evaluation, several sets of international regulatory guidelines were considered. Based on the number of parameters exceeding different guideline limits, the sum of the ranks was calculated and the relationship with the sediment characteristics known to affect metal concentrations (pH, organic content, chloride, and particle sizes) was examined.

Three regulatory guidelines are included for the total metal concentrations: (i) Swedish EPA guideline values for contaminated soil for less sensitive (LS) land use (SEPA 2016), (ii) values for sediment samples not classified as having good status (Class III-V) from Norwegian Environmental Agency Environmental Quality Standard (EQS) for contaminated sediments (Miljødirektoratet 2016), and (iii) two values, i.e., interim sediment quality guidelines (ISQGs) and probable effect levels (PELs) from Canadian Sediment Quality Guideline for the protection of freshwater aquatic life (CCME 2001) in which case both thresholds are used for the ranking and samples exceeding the lower threshold receive 0.5 rank while samples exceeding the upper threshold receive 1 rank. Table 2 shows the guideline values used for the ranking of the total concentrations and more information regarding the guidelines is provided in the Supplementary material.

**Table 2:** Guideline values used for the ranking of total, pore water, and DGT labile concentrations of metals in the pond sediment samples

Sediment/soil [mg/kg DW]					Water [µg/L]			
	Sweden^a^	Norway^b^	Canada^c^		France^d^	EU^e^	Sweden	
Metal	LS	EQS sediments (Class III)	ISQG	PEL	AA-EQS	AA-EQS	Range^f^	Calculated generic value for lakes^g^
Zn	500	139 (CW)	123	315	7.8		5.5–20	7.0
Cu	200	210 (FW)	35.7	197	1		0.5–12	2.4
Pb	400	66 (FW)	35	91.3		1.2	1.2–13	2.5
Ni	120	42 (CW)				4	4–16	8.4
Cr	150	112 (FW)	37.3	90	3.4			
Cd	12	1.5 (FW) for hardness < 40 mg CaCO_3_/L	0.6	3.5	5	0.08–0.25 (depends on water hardness (mg CaCO_3_ L^−1^))		

^a^Swedish EPA guideline value for less sensitive (LS) land use (Swedish EPA, 2016)

^b^Concentrations higher than Class II do not have good status. *CW* costal water, *FW* fresh waters (Miljødirektoratet 2016)

^c^Interim sediment quality guidelines (ISQGs) and probable effect levels (PELs) (CCME 2001)

^d^(Argilier et al. 2016)

^e^The EQS refer to bioavailable concentrations (EC 2013)

^f^Dissolved concentrations lower than the lower limit (“good” status) higher than the upper limit (“moderate” status for Cu and Zn and “not good” status for Ni and Pb) (HVMFS 2016)

^g^In case site-specific water data (pH, DOC, and Ca) are out of validated range for Bio-met (Bio-met 2015) in addition to comparison to bioavailable concentration dissolved concentration is also compared to the calculated generic value and the worse condition is chosen (HVMFS 2016)

For the ranking of both the pore water and DGT concentrations, three guidelines are used: (i) Swedish Agency for Marine and Water Management’s regulations for classification of surface water status (HVMFS 2016), (ii) European directive annual average environmental quality standards for surface fresh water (AA-EQS) (EC 2013), and (iii) French AA-EQS (Argilier et al. 2016). It should be noted that these guidelines are developed for water and not for the sediment pore water. Both EC (2013) and HVMFS (2016) consider metal’s bioavailability when estimating potential risks to the aquatic habitat (Table 2). The metal bioavailable concentrations are calculated using the Bio-met biotic ligand model based on pH, DOC [mg/L], and Ca [mg/L] measured in pore water in each sample. Since it is currently not recommended under the Water Framework Directive (WFD) to calculate bioavailable Pb EQS using Bio-met, this step is omitted in the case of Pb. More details on the method described in the HVMFS (2016) which was applied in this study can be found in the Supplementary material.

## Results and discussion

The presentation of the results starts with a short description of sediment characteristics, followed by metal concentrations from each method (total, pore water, DGT, sequential extraction, and toxicity). Finally, a section comparing different methods and parameters is presented.

### General parameters

Sediment characteristics (pH, cond, DO, DOC, Cl, TOC, LOI, N, C/N, %ClaySilt, and %Sand) for the individual samples are presented in Table S5 in the Supplementary material. pH values for the pond sediments were in the range 5.1–7.9 suggesting acidic and neutral, as well as alkaline pond sediment. These levels are comparable to pH values measured in 64 stormwater ponds across the USA 4.1–7.9 (Blaszczak et al. 2018). The most acidic samples were typically from Växjö (mean 5.8).

Measured LOI ranged from 1.5 to 35.9% DW which was lower than organic content measured in sediments from two ponds (66–74%) by Karlsson et al. (2010) and two pond inlets (39–52%) by Färm (2002). On the other hand, the measurements fall in the range of those observed by Blaszczak et al. (2018) (0.3–54.8% organic matter) but are higher compared to the levels observed by Mayer et al. (2008) (7.10% LOI). LOI positively correlated with TOC (*ρ* = 0.76, *p* = 4.46E-07) and N (*ρ* = 0.618, *p* = 1.62E-04) and negatively correlated with DO (*ρ* =  − 0.46, *p* = 0.008) (Fig. S6 in the Supplementary material).

Two samples (S5-O and S4-I) had high Cl (3420 and 1510 mg/kg DW) and conductivity (6180 and 3690 μS/cm) compared to the other samples (Table S5 in Supplementary material), probably due to the salt used for winter road maintenance, as both ponds have road catchments. High Cl concentrations (9–2921 mg/kg DW) were reported before in stormwater pond sediments (Blaszczak et al. 2018).

### Total metal concentrations

**Fig. 1:**
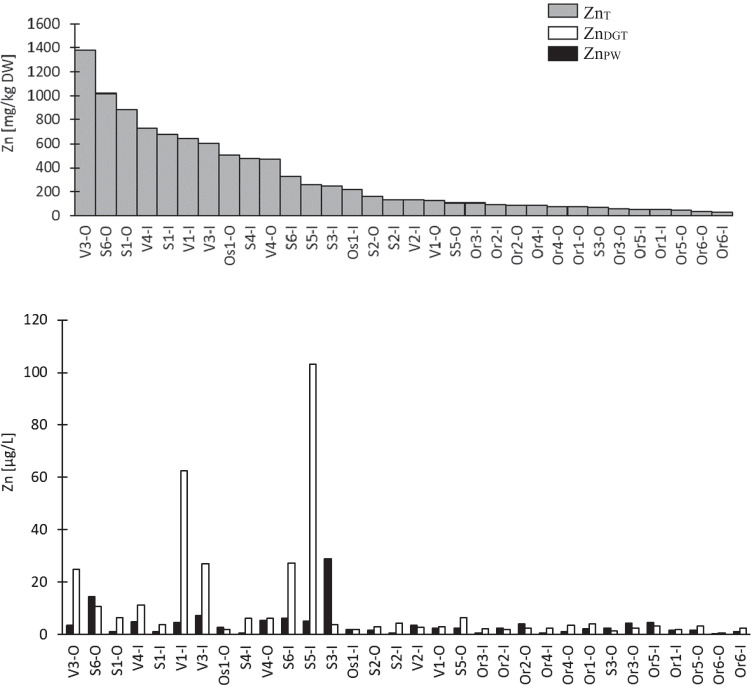
Top graph shows Zn_T_ concentrations ranked from highest to lowest. Bottom graph shows Zn_DGT_ and Zn_PW_ concentrations ranked according to total metal concentrations

**Fig. 2:**
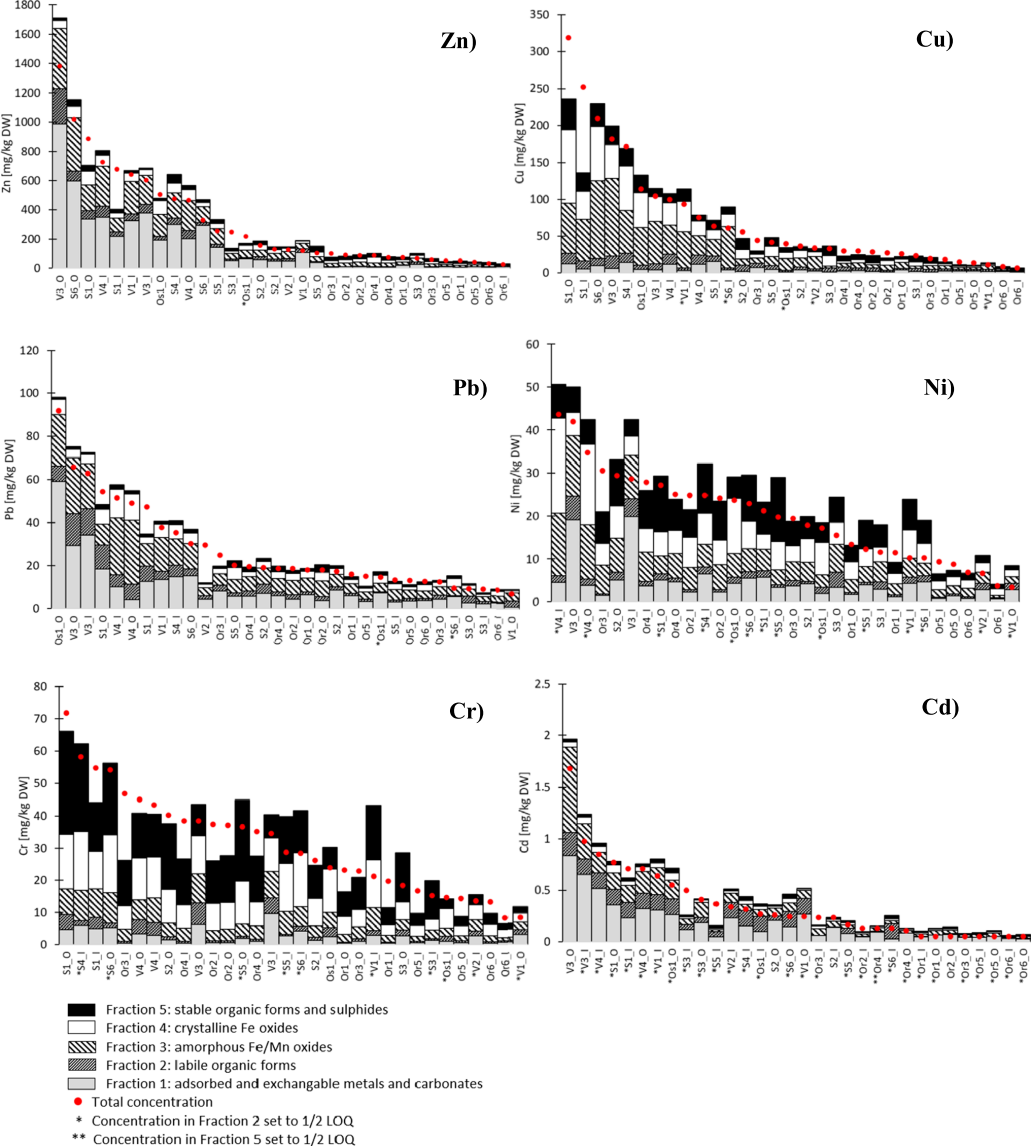
Total Zn, Cu, Pb, Ni, Cr, and Cd concentrations described with red dots and ranked from highest to lowest. Speciation of metals among the 5 fractions is described with stacked bars. Samples with concentrations < LOQ in Fraction 2 are marked with (*) and in case of Cd, one sample (Or4-I) had concentration in Fraction 5 < LOQ which is marked with (**)

Total metal concentrations varied among different ponds and between different metals. For instance, Zn_T_ concentrations were the highest of the 6 studied metals and ranged from 27.4 to 1380 mg/kg DW. Similarly, high variation was observed for Cu_T_ with maximum concentrations around 50 times higher than minimum concentrations (Table 3). Note that for assessing variability, samples that had concentrations < LOQ were set equal to LOQ. Due to this high variation, some samples from the Stockholm and Växjo ponds (Fig. 1) had higher concentrations than the maximum Zn_T_ and Cu_T_ concentrations reported in previous pond studies (Färm 2002; Marsalek et al. 2006; Karlsson et al. 2010; Frost et al. 2015). Cd_T_ concentrations (< 0.1–1.68 mg/kg DW) were the lowest (Table 3) and most comparable to levels detected in Swedish and Danish ponds 0.1–0.9 mg/kg DW (Färm 2002; Karlsson et al. 2010), whereas higher maximum concentrations (3.13 and 4.2 mg/kg DW) have been measured in other ponds around the world (Liebens 2001; Marsalek et al. 2006). Variation for Cr_T_, Ni_T_, and Pb_T_ was not that high; i.e., maximum concentration was 9 to 13 times higher than the minimum (Fig. 2 red dotes). Cr_T_ and Ni_T_ concentrations in 32 sediment samples are similar to the concentrations observed in 17 urban stormwater ponds by Frost et al. (2015). Pb_T_ concentrations fall in range reported previously 6.03–202 mg/kg DW (Färm 2002; Marsalek et al. 2006; Karlsson et al. 2010; Frost et al. 2015).

**Table 3:** General statistics (median and range) for total, pore water, and DGT labile metal concentrations. For censored data median values are calculated using Kaplan–Meier method in NADA

	Cd	Cr	Cu	Ni	Pb	Zn
Total concentration [mg/kg DW]						
Median	0.25	28.6	37.7	19.6	18.7	128
Range	< 0.1–1.68	8.44–71.9	6.14–319	3.4–43.6	7.00–91.8	27.4–1380
Pore water concentration [µg/L]						
Median	0.00538	0.971	0.626	2.63	0.164	2.40
Range	< 0.002–0.0383	0.0482–11.1	< 0.1–7.40	0.514–13.0	< 0.01–2.74	0.318–29.0
DGT labile concentration [µg/L]						
Median	0.00441	0.492	0.639	1.07	0.0753	3.51
Range	0.00094–0.0621	< 0.191–0.856	0.0592–4.97	0.261–5.18	0.0153–1.06	0.688–103

### Pore water metal concentrations

Dissolved metal concentrations consist of free ions, complexes with other inorganic ions (e.g., chloride), dissolved organic complexes (e.g., measured as DOC), and mineral colloids (Hin et al. 2010; Zhang and Davison 2015). Zn_PW_ concentrations in the 32 pond sediment samples are shown in Fig. 1. The maximum dissolved concentrations of Zn_PW_ (29 µg/L) and Cu_PW_ (7.4 µg/L) were measured in the same sample (S3-I). Pb_PW_ had the highest variation among the sample (< 0.01–2.74 µg/L) with the maximum concentration measured in the Or5-I sample. A high variation was also observed for Cr_PW_ (0.0482–11.1 µg/L) with the highest and the lowest concentrations measured in samples V4-O and Or1-I respectively. Maximum (13 µg/L) and minimum (0.514 µg/L) concentrations of Ni_PW_ were measured in samples V3-I and Or6-O respectively. Cd_PW_ had the lowest variation (< 0.002–0.0383 µg/L) among studied metals. Seven samples had one or more metals below LOQ: V1-O (Cu_PW_, Pb_PW_, and Cd_PW_), V1-I and V4-O (Cu_PW_ and Pb_PW_), S2-I (Cu_PW_ and Pb_PW_), S5-O (Cu_PW_), Or6-I and Or6-O (Cd_PW_).

A variety of methods have been used to extract pore water i.e. in laboratory (centrifugation (as in this study), pressurization or suction) and in situ (suction and “peepers”) (Burton Jr. 2010) which should be noted when comparing results. Dissolved concentrations observed in the pore water were relatively low compared to previous research. For example, metal concentrations in sediment pore water from a stormwater pond in Ontario were as follows: 15–60 µg/L (Zn_PW_), 4–11 µg/L (Cu_PW_), 7–110 µg/L (Cd_PW_), and 5–12 µg/L (Pb_PW_) (Mayer et al. 2008). Mayer et al. (2008) used dialysis membrane samplers deployed in the sediments to equilibrate with the sediment pore water for 2 weeks. Durin et al. (2007) also reported higher metal concentrations in sediment pore water: 220–980 µg/L (Zn_PW_), 20–157 µg/L (Cu_PW_), and 1–67 µg/L (Pb_PW_). In their study, eight sediment pore water samples were collected using lysimeter from a retention infiltration basin receiving highway runoff from a bridge (Durin et al. 2007).

### DGT labile concentration

DGT measurements give information on the concentrations of truly dissolved metals, metal complexes, and exchangeable metals from the solid phase (Table 1), which are able to accumulate on the binding layer, depending on their lability and diffusion coefficients (Van Leeuwen et al. 2005; Zhang and Davison 2015). DGT continuously removes dissolved metals from its deployment medium and gives “information on speciation in solution and solid-solution interactions in soils and sediments” (Zhang and Davison 2015). Figure 1 shows Zn_DGT_ concentrations in the 32 pond sediments. The maximum DGT concentrations for Zn and Cu were measured in the same sample (S5-I). This was the only sample where the surface of the sediment was above the water level which resulted in more oxygenated sediment, which is also reflected in higher DO level in this sample (4.19 mg/L) compared with the rest of the samples (< 0.001–0.459 mg/L), as well as the sample with the lowest proportion of fine (clay and silt) particles. Zn_DGT_ had the highest variation among the pond samples (0.688–103 µg/L), followed by Cu_DGT_ (0.0592–4.97 µg/L). DGT concentrations had the lowest variation in the case of Cr (< 0.191–0.856 µg/L), with a maximum concentration 4.5 times higher than the minimum. To the authors’ knowledge, no previous studies have used DGT on stormwater pond sediments.

### Sequential extraction

Sequential extraction is used to investigate the potential for metals to be released from sediments due to changes in the environmental conditions. Figure 2 shows total concentrations and speciation of the six studied metals. Zn, Pb, and Cd had similar speciation with high concentrations in Fraction 1 (adsorbed and exchangeable metals and carbonates). In comparison, speciation of Cu differed with a more pronounced share of the last two fractions (crystalline Fe, as well as stable organic forms and sulfides). Lastly, Cr and Ni had a higher abundance in Fraction 5 as compared to other metals.

These results are in agreement with previous research, although some uncertainty is inherent to this comparison of results due to the use of different speciation schemes. Karlsson et al. (2016) used the five-step sequential extraction from Tessier et al. (1979) and found Pb, Cd, and Zn mainly bond to first three fractions, Cu mainly bound to organic matter (Fraction 4) while Cr and Ni were mainly in Fraction 5 (> 70% and > 60% respectively). Mayer et al. (2008) also used the Tessier et al. (1979) extraction procedure and reported the highest abundance of Cd in the (exchangeable) Fraction 1, while for Cu the highest abundance (46%) was in organic matter (Fraction 4) (Mayer et al. 2008). Durand et al. (2004) analyzed the speciation of Cd, Zn, Ni, Cr, Pb, and Cu using Community Bureau of Reference (BCR) Sequential Extraction. Cd was the most abundant in the exchangeable fraction and Cu in the organic fraction (60–80%) while Ni (60–90%) and Cr (70–85%) were concentrated in the last step (Fraction 4) (Durand et al. 2004).

### Toxicity

For most samples, the toxic response in the Microtox test was too low to allow the calculation of effective concentration (EC) values. EC20 and EC50 are the concentrations of the sample in the suspension (ml/L) producing a 20% and 50% decrease in luminescence compared to the control sample. EC20 values (10.1–708 ml/L) were reported for five (Or6-O, S3-I, S3-O, V1-O, V4-O) out of 32 samples. Of these five, three samples (S3-O, V1-O, V4-O) also had EC50 reported in a range 24–792 ml/L.

The aim of this study was to consider a method for toxicity evaluation that is common in practice and to evaluate the information relative to results from other metal analyses. It should be noted that while Microtox has the advantage of being a simple, standardized procedure to screen for toxicity, the absence of toxicity through the Microtox test should not be interpreted as a lack of toxicity to any organism. For example, a battery of five tests was used for toxicity testing which showed Microtox to be the least sensitive (Marsalek et al. 1999). Using Microtox along with other toxicity tests, simultaneously or sequentially, is generally recommended for environmental assessment of sediments (Doherty 2001).

Total metal concentrations, pore water, and DGT labile concentrations in the 5 samples with EC20 values reported were not significantly different than those in the remaining samples (Table S6 in Supplementary material). Scholes et al. (2007) used Microtox to investigate the toxicity of surface water, resuspension water (i.e., water sample in which sediments had been resuspended), pore water, and sediment samples collected along an urban watercourse in London. The authors found some correlations between toxicity and metal concentrations, but, as in the present study, observed no significant differences between the metal levels when samples were divided into two subsets (with and without toxicity reported) and their metal levels compared.

### Correlation and comparison between the methods and parameters

#### PCA

**Fig. 3:**
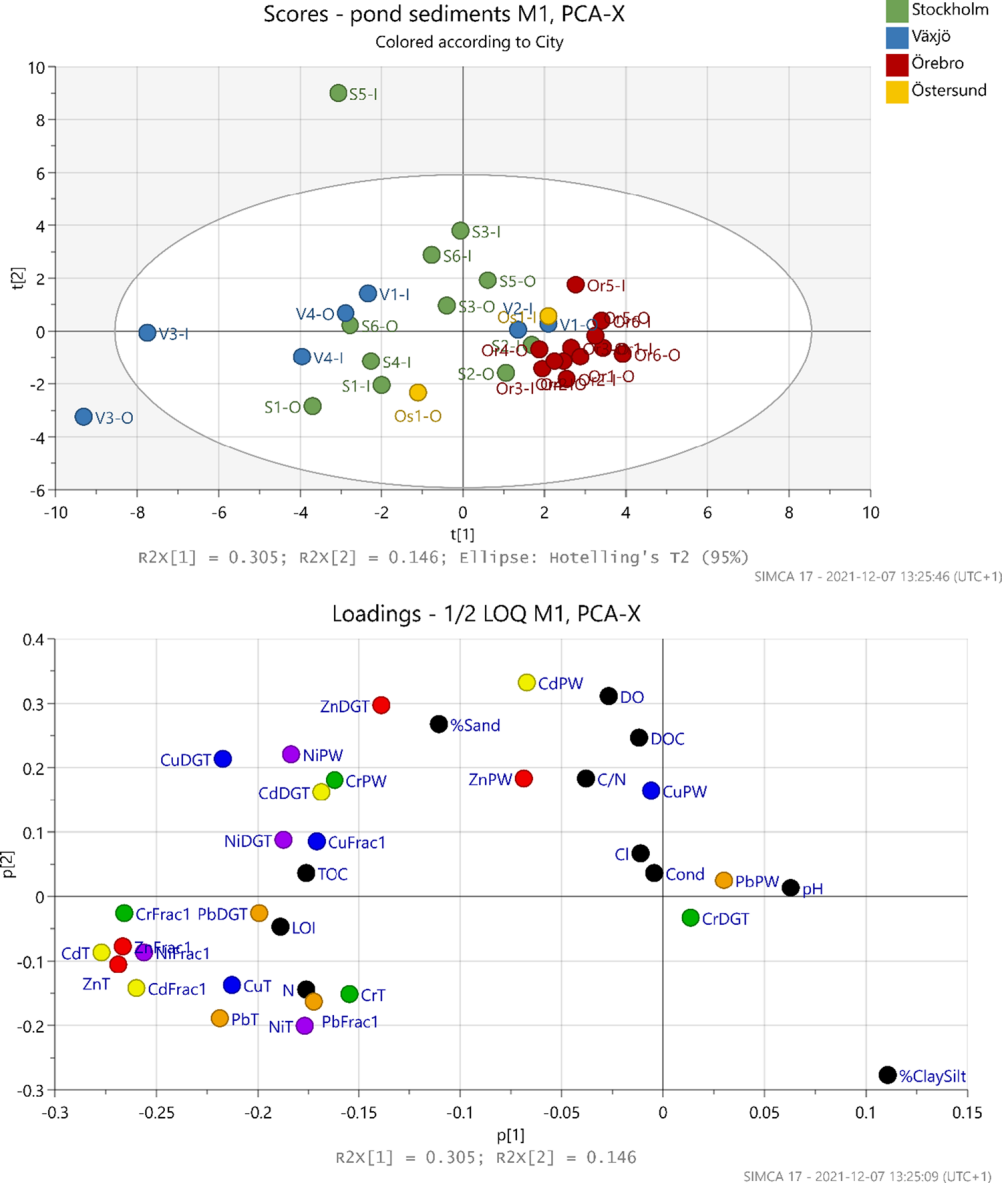
Score plot (top panel) and loading plot (bottom panel) for PCA where values < LOQ are replaced with ½ LOQ. Labels on score plot indicate sample names and coloring is done based on different cities. In the loading plot, labels indicate different methods and methods explaining the same metal are colored with the same color. General parameters are colored in black

In the PCA, sediment characteristics were summarized in seven components explaining 81.5% of the data variation, where the first and second component explained 30.5% and 14.6%, respectively (Fig. 3). The variables city and catchment type were set as secondary identifiers and grouping by city showed a clearer grouping of the samples (Fig. 3) compared to grouping by catchment type (Fig. S1 Supplementary material). For example, all total concentrations are opposite to Örebro samples suggesting the lower concentrations in Örebro whereas no clear relationship is found for different catchment types since samples with the highest and lowest total concentrations are both from industrial and/or commercial catchments (Fig. S1 Supplementary material).

Finally, examining the PCA score plot (Fig. 3 upper panel) shows that Örebro ponds clearly clustered together confirming what was previously seen in Fig. 1, i.e., little variation in metal concentrations between the different Örebro ponds. This separation of Örebro samples was also observed in a study of organic pollutants for the same samples (Flanagan et al. 2021). Moreover, total metals in general are located mostly to the left (PCA loading plot) but not in a very clear cluster, and coloring different analyses (of the same metal) shows no clear grouping, except to some extent for total metals and Fraction 1 (i.e., these two methods are grouped together in the cases of Zn, Cd, and Pb). The implication of this is that the metals do not have a consistent speciation between ponds. This could be due to differences in the characteristics of sediment between ponds that influence metal fate (e.g., particle size, pH, or organic content) or due to different sources (e.g., traffic, industry) between catchments. Possible specific correlations between different methods are further examined in the following sections through scatter plots and correlation coefficients.

#### Total metals and Fraction 1

**Fig. 4:**
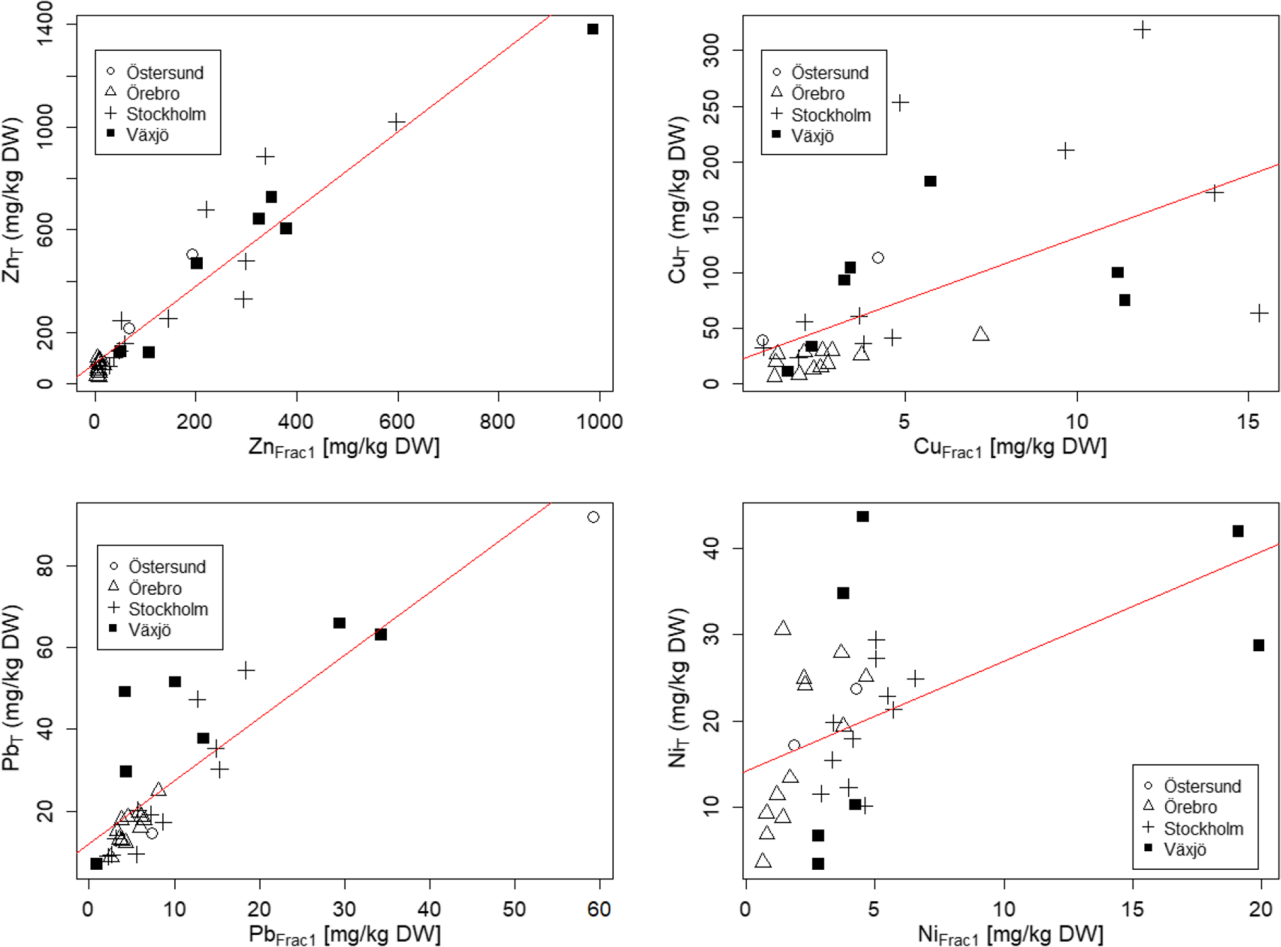
All values were above LOQ. Noncensored scatterplot shows Fraction 1 and total concentrations for Zn (top left), Cu (top right), Pb (bottom left), and Ni (bottom right) where observations are grouped by city

The PCA loading plot (Fig. 3) indicated some correlations between total metals and Fraction 1 of the sequential extraction. Thus, correlation tests were conducted and a significant positive correlation for all metals except Cr was found (Table S7 in Supplementary material). Correlation analysis between total metals and other extraction steps showed significant correlations, with some exceptions (Table S8 in the Supplementary material). Although there is an indication of positive correlations (Fig. 4) that the higher total concentrations correspond to higher (potential) risk, the strength of this relationship varied between the metals and in the case of Cu, Ni, and Cr, total concentrations would not be enough to infer about the potential risk. The same was true when inlet and outlet samples were considered separately; i.e., Zn and Pb had the strongest correlations between total concentrations and fraction 1 (ρ> 0.80) whereas for other metals the strength of the relationship varied and would not be enough to infer about the potential risk.

#### Pore water and DGT

**Fig. 5:**
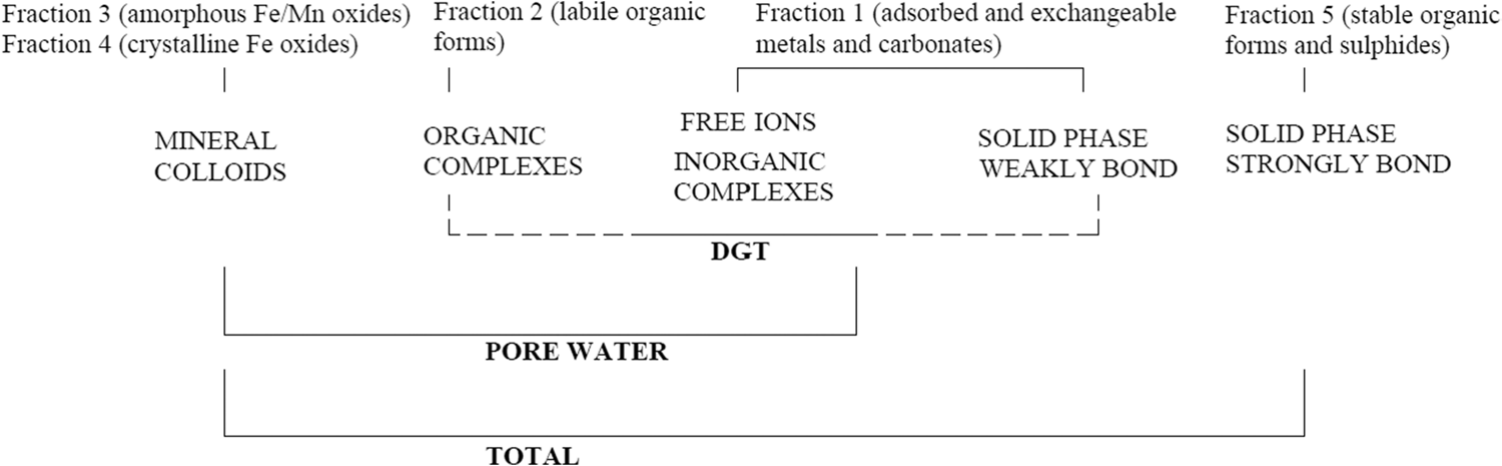
Different methods and measurable metal fractions (inspired by Zhang and Davison (2015))

As previously mentioned, pore water metal concentrations include free ions, as well as complexes and colloids, while DGT measurements describe labile metals that may be in the dissolved phase or easily mobilized from the solid phase (Fig. 5).

Here we evaluate the hypothesis that the two methods are correlated, which would imply that the dissolved labile phase is dominant or has a consistent relationship with other dissolved species (mineral colloids, complexes partially measured by DGT) and the solid labile fraction. For Pb, Ni, and Cr, the maximum and median concentrations in pore water were higher than the maximum and median DGT concentrations (Table 3). This could mean that metals bonded to mineral colloids and organic complexes (not detected by DGT) represent significant portions and that the contribution from the solid phase pool to DGT labile concentrations was in higher proportions in case of Zn, Cu, and Cd. The PCA loading plot did not indicate any strong relationship between pore water concentrations and DGT (Fig. 3). When examining correlations between the two methods, only Ni correlated strongly (*ρ* = 0.682 and *p* = 2.78E-05) (Fig. S4 in the Supplementary material), which was also true when correlations were tested separately for inlet and outlet samples (*ρ* = 0.7 and *p* < 0.01). As such, it cannot be concluded that the two methods can be used interchangeably for all metals; rather, they complement each other by contributing with different information about the sediments.

#### Fraction 1 of sequential extraction and DGT

DGT measurements can include exchangeable metals from the solid phase whereas metals strongly bound to the solid phase are not measured (Zhang and Davison 2015). As shown in Table 1, Fraction 1 measures adsorbed and exchangeable metals and carbonates; thus, a potential correlation between DGT and the first fraction is investigated. This correlation would indicate that the contributions of the metals from the solid phase to DGT are dominant or have a consistent relationship with the solid labile fraction. In many cases, metal concentrations in Fraction 2 were < LOQ (indicated with a symbol (*) next to the sample name in Fig. 2). For this reason, the correlation analysis was limited to Fraction 1, although part of the Fraction 2 fraction (when present) may also contribute to DGT labile concentrations.

Although the PCA loading plot did not suggest any strong correlations between the Fraction 1 and DGT methods (Fig. 3), significant correlations were found for Zn (*ρ* = 0.714 and *p* = 8.50E-06) and Cu (*ρ* = 0.637 and *p* = 8.75E-05). However, the correlation does not appear very clear, even when samples are grouped according to city (Fig. S5 in the Supplementary material) or according to location in the pond. When inlet and outlet samples are considered separately, significant correlations were observed for Zn and Cu for inlet samples but not for outlet samples, for which the two methods appeared correlated from the scatterplot but not according to the statistical test (*p* = 0.014 and *p* = 0.025, for Zn and Cu respectively). As in the previous section (comparison between pore water and DGT), the results do not allow for a generalization that the two methods could be substituted.

#### Correlation with general parameters

One purpose of this analysis was to investigate if any of the general parameters can be indicators for metals or their bioavailability.

LOI had significant correlations (*p* < 0.01) with total metals: Zn_T_, Cu_T_, and Cd_T_ (Table S9 in the Supplementary material). Positive correlations were found between total metals (Cd, Pb, Cu, Zn, Mo) in pond sediments and organic carbon content measured by LOI which may indicate the importance of adsorption to organic matter (Frost et al. 2015) and/or that the metals have similar sources with organic matter (for example, tire particles). To further examine this effect, correlations between partition coefficients (K_d_, the ratio of total solid concentration to dissolved pore water concentrations) for metals and organic matter were also examined, which generally did not show the positive correlations that would be expected if organic matter did increase sorption capacity. Indeed, significant negative correlations were found in the cases of the following: Cr_Kd_ and TOC (rho =  − 0.488, *p* = 0.005), Cr_Kd_ and LOI (rho =  − 0.524, *p* = 0.002), and Cr_Kd_ and DOC (rho =  − 0.493, *p* = 0.005), which can be explained by LOI, TOC, and DOC having significant positive correlation with Cr_D_. The only positive correlation was observed between Cd_Kd_ and LOI (rho = 0.485, *p* = 0.005), for which the relation was not very clear and the presence of a relatively high proportion of censored data may have biased the result of the correlation test. Overall, this analysis therefore supports the hypothesis that the positive correlations between organic matter and total metal concentrations are due to a source of particles in urban runoff that contain both metals and organic matter (e.g., tire/road wear particles) rather than an influence of organic matter on the sorption of dissolved metals.

Since the organic carbon content of sediment can influence the bioavailability of sediment-associated contaminants, the correlation between TOC and Fraction 2 was investigated and significant correlations were found (tau = 0.415–0.558) except for Ni_Frac2_ and Cd_Frac2_ (Table S10 in the Supplementary material). It should be noted that for Cr_Frac2_ 31% of the values were < LOQ; however, examining the censored scatterplot, there is an indication of positive correlation, which was not the case for Ni_Frac2_ and Cd_Frac2_ (44 and 84% censored values respectively). The dominant size fraction (mean = 74%) was silt and clay fraction (≤ 63 µm), while the share of sand fraction (≤ 2000 µm) for 32 samples was 26% (mean value). The sample with the lowest clay and silt fraction was S5-I (22%) while the sample with the highest fraction (100%) was Or2-I. On average, samples from Örebro had a higher share of fine particles (89%) compared to other cities (61–75%). This is also indicated in the PCA score plot, where the Örebro samples are grouped on the right side of the PCA score plot together with the %ClaySilt parameter (Fig. 3). This is in contrast to what would be expected, i.e., higher metal concentrations in the finer material (Liebens 2001; Hilliges et al. 2017). For example, a positive correlation between the percentage of silt and clay particles (particles < 63 µm) and total metals (most notably for Zn and to lower extent also Cu, Ni, and Cr) were reported for pond sediment by Karlsson et al. (2010). No positive correlation was observed between total metals and the fraction of silt and clay in our dataset. This indicates that the particle size (and thus ability to adsorb metals) is not the major determining factor of metal concentrations, possibly because some sources of metals in the urban environment are particulate (e.g., tire and road wear particles), rather than sources of dissolved metals which adsorb to particles. In the case of Örebro, where four facilities (Or-2, Or-4, Or-5, and Or-6) received water through open channels and the catchments of two facilities (Or-1 and Or-3) had relatively high proportions of permeable surfaces, particles from eroded permeable surfaces may have diluted particles originating from impervious urban surfaces (Flanagan et al. 2021) which resulted in the least polluted samples (Örebro) having the highest fraction of clay and silt. Moreover, pH affects metal mobility (Rieuwerts et al. 1998); thus, statistical tests were performed that showed significant correlation only between pH and Zn_PW_ (*ρ* =  − 0.466, *p* = 0.007) and Pb_PW_ (tau =  − 0.337, *p* = 0.007). However, when exploring the scatterplots, the correlation did not appear clear (Fig. S7 in Supplementary material).

#### Ranking table

Table 4 shows the ranks of 32 samples in descending order. These ranks can be seen as an overall evaluation of environmental risk due to the contamination of each sample by trace metals, incorporating different metals, different speciations, and different ecological and/or human health endpoints. Although S6-O and V3-I were not the most polluted samples when looking at the total metal concentrations (except for Zn), Table 4 shows that they had the highest frequency of exceeding the guidelines (∑ranks = 13.5 and 12.5 respectively) and thus were top-ranked. In fact, the first 11 ranked samples (∑ranks > 5.5) were all from Stockholm and Växjö and exceeded two or more guidelines in two or more categories (Table 4). None of the Örebro samples was found to have high ranks, which supports what was previously shown in the PCA score plot (clear clustering of the Örebro samples in the opposite side of the PCA origin compared to metal concentrations).

**Table 4:** Ranking table for pond sediments for 6 metals and their total, pore water, and DGT labile concentrations as well as Microtox EC20 (*) results. If the metal is listed under specific guideline it means that its respective concentration in the sample in question exceeded the guidline value

Sample name	Total concentrations				Pore water			DGT labile concentrations			Microtox EC20	∑Ranks
	Sweden^a^	Norway^b^	Canada^c^		France^d^	EU^e^	Sweden^f^	France^d^	EU^e^	Sweden^f^		
			ISQG	PEL								
S6-O	Cu, Zn	Zn	Cr	Cu, Zn	Cr, Cu, Zn		Cu, Zn	Cu, Zn		Zn		13.5
V3-I	Zn	Zn	Cd, Cu, Pb	Zn	Cr, Cu, Zn		Ni	Cu, Zn		Cu, Zn		12.5
V4-I	Zn	Ni, Zn	Cd, Cr, Cu, Pb	Zn	Cr			Cu, Zn		Zn		10.0
V3-O	Zn	Cd, Zn	Cd, Cr, Cu, Pb	Zn				Cu, Zn		Cu, Zn		10.0
S5-I		Zn	Cu, Zn		Cr, Cu		Ni	Cu, Zn		Cu, Zn		9.0
S1-I	Cu, Zn	Cu, Zn	Cd, Cr, Pb	Cu, Zn				Cu				8.5
S1-O	Cu, Zn	Cu, Zn	Cd, Cr, Pb	Cu, Zn				Cu				8.5
V1-I	Zn	Cu, Zn	Cd, Cu, Pb	Zn				Cu, Zn		Zn		8.5
S3-I		Zn	Zn		Cr, Cu, Zn		Cu, Zn				*	7.5
V4-O		Zn	Cd, Cr, Cu, Pb	Zn	Cr		Ni				*	7.0
S6-I		Zn	Cu	Zn	Cr			Cu, Zn		Zn		6.5
Os1-O	Zn	Pb, Zn	Cu	Pb, Zn								5.5
S4-I		Zn	Cr, Cu, Pb	Zn				Cu				4.5
S3-O					Cr, Cu						*	3.0
S2-O		Zn	Cr, Cu, Zn									2.5
S5-O			Cu		Cr			Cu				2.5
Or5-I					Cu		Cu					2.0
Os1-I		Zn	Cu, Zn									2.0
Or3-I			Cr, Cu		Cu							2.0
Or4-O					Cu		Cu					2.0
S2-I			Cu, Zn									1.0
Or2-O					Cu							1.0
Or3-O					Cu							1.0
Or6-O											*	1.0
V1-O											*	1.0
Or2-I			Cr									0.5
Or4-I			Cr									0.5
V2-I			Zn									0.5
Or5-O												0
Or1-I												0
Or1-O												0
Or6-I												0

^a^Swedish EPA guideline value for less sensitive land use (Swedish EPA, 2016)

^b^Norwegian Environmental Agency Environmental Quality Standard for contaminated sediments (Miljødirektoratet 2016)

^c^Canadian Sediment Quality Guideline for the protection of freshwater aquatic life (CCME 2001). If total concentrations exceed interim sediment quality guidelines (ISQG), 0.5 rank is given; if probable effect levels (PEL) are exceeded, rank 1 is given

^d^French annual average environmental quality standards for surface fresh water (Argilier et al. 2016)

^e^European directive annual average environmental quality standards for surface fresh water (EC 2013)

^f^ (HVMFS 2016)

Correlations between the ranks and the sediment characteristics (which are known to affect metal adsorptions such as pH, organic content, chloride, and particle sizes) showed a significant positive correlation with LOI (rho = 0.68, *p* = 2.064e-05).

**Fig. 6:**
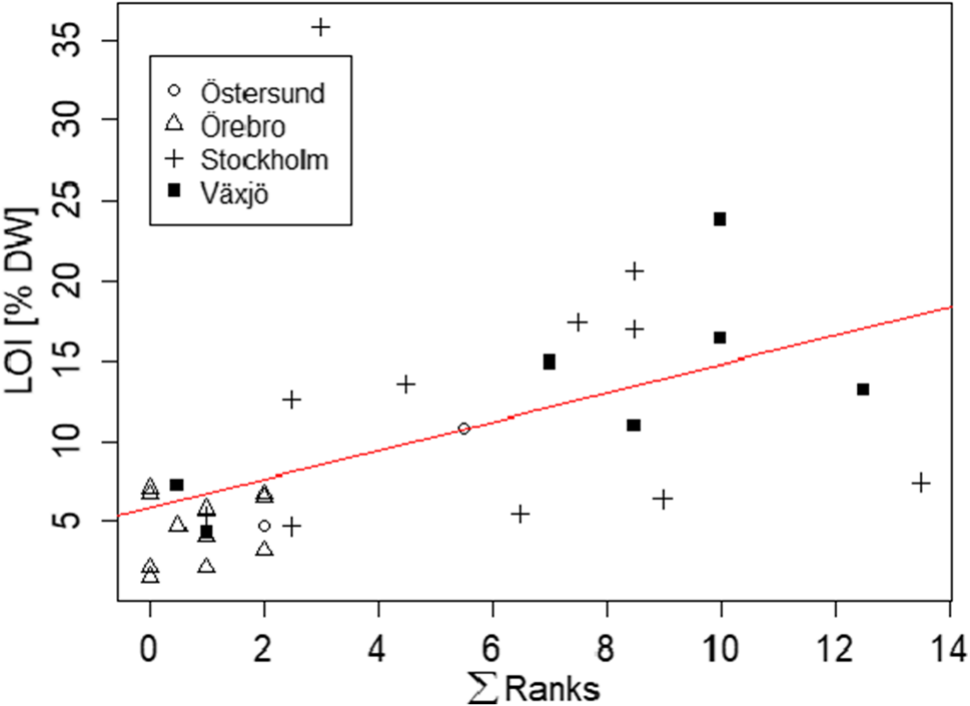
Relationship between LOI and ranks where observations are grouped by city

The lowest values for LOI (1.5% DW) were measured in an Örebro sample (Or1-I) and Örebro had the lowest average of LOI (4.6% DW) compared to other cities (LOI = 7.8–13.3% DW). Examining scatterplot between ranks and LOI (Fig. 6) along with the ranking table (Table 4) shows that top ranked samples (S6-O and V3-I) had lower LOI (7.4 and 13.2% DW respectively) compared to, for example, sample S3-O with the highest LOI (35.9% DW) and rather low rank (∑ranks = 3). During sampling, S3-O was observed to be notably different in composition, i.e., mainly peat with a thin black (sludge) layer. It is likely that this natural organic material (not polluted by metals) contributed to high LOI in the case of S3-O. This means that while high LOI can generally correspond to high total metal levels, there is no perfect relationship and it cannot be seen as a surrogate to metal analysis and overall environmental risk assessment of stormwater pond sediments.

The sensitivity of the analysis to indicate problematic sediments decreased in the following order: total concentrations (21 samples detected) > pore water concentrations (14) > DGT labile concentrations (11) > Microtox (5). In some samples, total concentrations as well as DGT labile concentrations and/or pore water concentrations of the same metal exceeded certain regulatory guideline value. This can give an indication of a higher risk for the pond ecosystem because in addition to the metal burdens being high, they are or can also become mobile/bioavailable. Moreover, some management practices such as de-watering should be avoided due to the risk of metal release to the water phase (Karlsson et al. 2016). Thus, total concentrations should be part of risk assessment of the sediments, with other methods used as complementary methods. When it comes to total concentrations of Zn, Cu, Pb, Cd, and Cr (whose threshold limits are available from all three guidelines (Table 2)), frequency of exceeding the total concentration guidelines decreases in the following order: Zn (40) < Cu (23) < Cr (11) < Pb (9) < Cd (8). As stated before, Microtox was not sensitive enough to point to the most problematic sediments with respect to metals.

## Conclusions

In this study, sediment was sampled from inlets and outlets of 17 stormwater sedimentation facilities and analyzed with a combination of different methods to evaluate and discuss the comparability of methods and the suitability of each method to facilitate proper maintenance of stormwater ponds.

DGT labile concentrations were applied for the first time on stormwater pond sediments to examine comparability with the dissolved metal fraction in pore water and metal fraction from the first extraction step of sequential extraction. This comparison did not allow for generalization that the DGT could be substitute to either method. No clear clustering (PCA loading plot) between different methods (except to some extent for total metal fraction and fractions from sequential analysis) was observed probably because of large variability in the speciation of the same metal across different ponds.

The ponds in the city of Örebro were identified to have lower metal pollution both by the PCA and the overall environmental risk assessment (ranking table). This was attributed to the dilution of anthropogenic metal sources by particles from erosion of permeable catchment areas and open channels carrying stormwater to the ponds.

Most of the sediment samples were not acutely toxic with respect to V. Ficheri which was shown by very few samples with inhibition > 20%. Also, no significant difference in metal concentrations (total, pore water, and DGT) was observed between the samples that had EC values reported compared to the rest of the samples.

A significant positive correlation was observed between the frequency of sediment sample exceedance of guidelines (∑ranks) and LOI, indicating that high metal levels correlate to high LOI values. This appears to be due to LOI and metals having similar sources (e.g., tire/road wear particles) rather than by the ability of organic material (estimated by LOI) to increase the sorption of metals, as organic matter generally did not correlate positively with metal partition coefficients. However, the correlation of LOI and metals does not suffice to generalize that LOI can be seen as a surrogate to metal analysis because the relationship was not perfect (Spearman rho = 0.68, *p* = 2.064E-05).

In conclusion, high metal variability across different ponds (e.g., Zn_T_ and Cu_T_) means that the risk associated with stormwater pond sediments differs between sites and that a comprehensive analysis of different parameters is needed to better understand metal speciation and bioavailability and plan for proper maintenance.

## Supplementary Information

Below is the link to the electronic supplementary material.

Supplementary file1 (DOCX 380 KB)

## Data Availability

The data is available in the Supplementary material.

## References

[CR1] Al-Rubaei AM, Merriman LS, Hunt WF (2017). Survey of the Operational Status of 25 Swedish Municipal Stormwater Management Ponds. J Environ Eng.

[CR2] Argilier C, Augeard B, Baudoin JM, Beaudelain Poulain P, Beaujeu G et al (2016) Guide technique relatif à l’évaluation de l’état des eaux de surface continentales (cours d’eau, canaux, plans d’eau) (Technical guide to assessing the state of continental surface waters (water streams, canals, bodies of water)) (In French). Available at: https://hal.inrae.fr/hal-02603509 (Last visited on April 29, 2021)

[CR3] Bio-met (2015) Technical guidance to implement bioavailability-based environmental quality standards for metals.

[CR4] Becouze-Lareure C, Dembélé A, Coquery M (2019). Assessment of 34 dissolved and particulate organic and metallic micropollutants discharged at the outlet of two contrasted urban catchments. Sci Total Environ.

[CR5] Blaszczak JR, Steele MK, Badgley BD, Heffernan JB, Hobbie SE, Morse JL, Rivers EN, Hall SJ, Neill C, Pataki DE, Groffman PM, Bernhardt ES (2018). Sediment chemistry of urban stormwater ponds and controls on denitrification. Ecosphere.

[CR6] Blecken GT, Hunt WF, Al-Rubaei AM, Viklander M, Lord WG (2017). Stormwater control measure (SCM) maintenance considerations to ensure designed functionality. Urban Water J.

[CR7] Blecken GT, Rentz R, Malmgren C, Öhlander B, Viklander M (2012). Stormwater impact on urban waterways in a cold climate: variations in sediment metal concentrations due to untreated snowmelt discharge. J Soils Sediments.

[CR8] Brudler S, Rygaard M, Arnbjerg-nielsen K, Zwicky M, Ammitsøe C, Vezzaro L (2019). Pollution levels of stormwater discharges and resulting environmental impacts. Sci Total Environ.

[CR9] Burton GA (2010). Metal bioavailability and toxicity in sediments. Crit Rev Environ Sci Technol.

[CR10] Canadian Council of Ministers of the Environment (CCME) (2001) Canadian sediment quality guidelines for the protection of aquatic life: Summary tables. Updated. In: Canadian environmental quality guidelines, 1999, Canadian Council of Ministers of the Environment, Winnipeg. https://ccme.ca/en/resources/sediment# (accessed 7 Dec 2021).

[CR11] Degryse F, Smolders E, Oliver I, Zhang H (2003). Relating soil solution Zn concentration to diffusive gradients in thin films measurements in contaminated soils. Environ Sci Technol.

[CR12] Doherty, F.G., 2001. A review of the Microtox® toxicity test system for. Water Qual. Res. J. Canada 36, 475–518. 10.2166/wqrj.2001.027

[CR13] Drake J, Guo Y (2008). Maintenance of wet stormwater ponds in Ontario. Can Water Resour J.

[CR14] Dunn RJK, Teasdale PR, Warnken J, Jordan MA, Arthur JM (2007). Evaluation of the in situ, time-integrated DGT technique by monitoring changes in heavy metal concentrations in estuarine waters. Environ Pollut.

[CR15] Durand C, Ruban V, Amblès A (2004). Mobility of trace metals in retention pond sediments. Environ Technol.

[CR16] Durin B, Béchet B, Legret M, Le Cloirec P (2007). Role of colloids in heavy metal transfer through a retention-infiltration basin. Water Sci Technol.

[CR17] EC (2013) Directive 2013/39/EU of the European Parliament and of the Council of 12 August 2013 Amending Directives 2000/60/EC and 2008/105/EC as Regards Priority Substances in the Field of Water Policy

[CR18] EN 12176 (1998) Characterization of sludge - determination of pH-value. European Committee for Standardization (CEN)

[CR19] Eriksson E, Baun A, Scholes L (2007). Selected stormwater priority pollutants - a European perspective. Sci Total Environ.

[CR20] Eriksson, L., Byrne, T., Johansson, E., Trygg, J., Vikström, C., 2013. Multi- and megavariate data analysis. Basic Principles and Applications, Third revi. ed. MKS Umetrics AB, Malmö, Sweden.

[CR21] Färm C (2002). Evaluation of the accumulation of sediment and heavy metals in a storm-water detention pond. Water Sci Technol.

[CR22] Flanagan K, Blecken GT, Österlund H, Nordqvist K, Viklander M (2021). Contamination of urban stormwater pond sediments: a study of 259 legacy and contemporary organic substances. Environ Sci Technol.

[CR23] Frost PC, Song K, Buttle JM, Marsalek J, McDonald A, Xenopoulos MA (2015). Urban biogeochemistry of trace elements: what can the sediments of stormwater ponds tell us?. Urban Ecosyst.

[CR24] Hall GEM, Vaive JE, Beer R, Hoashi M (1996). Selective leaches revisited, with emphasis on the amorphous Fe oxyhydroxide phase extraction. J Geochemical Explor.

[CR25] Hall GEM, Vaive JE, MacLaurin AI (1996). Analytical aspects of the application of sodium pyrophosphate reagent in the specific extraction of the labile organic component of humus and soils. J Geo.

[CR26] Han L, Zhao X, Jin J, Gao B, Yang Y, Sun K, Li F (2019). Using sequential extraction and DGT techniques to assess the efficacy of plant- and manure-derived hydrochar and pyrochar for alleviating the bioavailability of Cd in soils. Sci Total Environ.

[CR27] Hayman NT, Rosen G, Strivens JE (2019). Evaluating the efficacy of DGT to quantify copper in stormwater at end-of-pipe. Chemosphere.

[CR28] Hilliges R, Endres M, Tiffert A, Brenner E, Marks T (2017). Characterization of road runoff with regard to seasonal variations, particle size distribution and the correlation of fine particles and pollutants. Water Sci Technol.

[CR29] Hin JA, Osté LA, Schmidt CA (2010) Guidance document for sediment assessment. Methods to determine to what extent the realization of water quality objectives of a water system is impeded by contaminated sediments, Ministry of Infrastructure and the Environment - DG Water

[CR30] Huber M, Welker A, Helmreich B (2016). Critical review of heavy metal pollution of traffic area runoff: occurrence, influencing factors, and partitioning. Sci Total Environ.

[CR31] HVMFS (2016) Miljögifter i vatten - klassificering av ytvattenstatus (Environmental toxins in water - classification of surface water status). (In Swedish). Havs- och vattenmyndighetens rapport. Göteborg. https://www.havochvatten.se/data-kartor-och-rapporter/rapporter-och-andra-publikationer/publikationer/2016-12-19-miljogifter-i-ytvatten---klassificering-av-status.html (accessed 13 Aug 2021)

[CR32] Karlsson K, Blecken G-T, Öhlander B, Viklander M (2016). Environmental risk assessment of sediments deposited in stormwater treatment facilities: trace metal fractionation and its implication for sediment management. J Environ Eng.

[CR33] Karlsson K, Viklander M, Scholes L, Revitt M (2010). Heavy metal concentrations and toxicity in water and sediment from stormwater ponds and sedimentation tanks. J Hazard Mater.

[CR34] Karouna-Renier NK, Sparling DW (1997). Toxicity of stormwater treatment pond sediments to Hyalella azteca (Amphipoda). Bull Environ Contam Toxicol.

[CR35] Kreuzeder A, Santner J, Zhang H (2015). Uncertainty evaluation of the diffusive gradients in thin films technique. Environ Sci Technol.

[CR36] Liebens J (2001). Heavy metal contamination of sediments in stormwater management systems: the effect of land use, particle size, and age. Environ Geol.

[CR37] Manzano R, Rosende M, Leza A, Esteban E, Peñalosa JM, Miró M, Moreno-Jiménez E (2019). Complementary assessment of As, Cu and Zn environmental availability in a stabilised contaminated soil using large-bore column leaching, automatic microcolumn extraction and DGT analysis. Sci Total Environ.

[CR38] Marsalek J, Marsalek M (1997). Characteristics of sediments from a strormwater management pond. Wat Sci Tech.

[CR39] Marsalek J, Brownlee B, Mayer T (1997). Heavy Metals and PAHs in Stormwater Runoff from the Skyway Bridge, Burlington, Ontario. Water Qual Res J Canada.

[CR40] Marsalek J, Rochfort Q, Mayer T, Servos M, Dutka B, Brownlee B (1999). Toxicity testing for controlling urban wet-weather pollution: advantages and limitations. Urban Water.

[CR41] Marsalek J, Watt WE, Anderson BC (2006). Trace metal levels in sediments deposited in urban stormwater management facilities. Water Sci Technol.

[CR42] Martin JM, Nirel P, Thomas AJ (1987). Sequential extraction techniques: promises and problems. Mar Chem.

[CR43] Mayer T, Rochfort Q, Borgmann U, Snodgrass W (2008). Geochemistry and toxicity of sediment porewater in a salt-impacted urban stormwater detention pond. Environ Pollut.

[CR44] McDonald S, Holland A, Simpson SL (2022). Metal forms and dynamics in urban stormwater runoff: new insights from diffusive gradients in thin-films (DGT) measurements. Water Res.

[CR45] Meylan S, Odzak N, Behra R, Sigg L (2004). Speciation of copper and zinc in natural freshwater: Comparison of voltammetric measurements, diffusive gradients in thin films (DGT) and chemical equilibrium models. Anal Chim Acta.

[CR46] Miljødirektoratet (2016) Grenseverdier for klassifisering av vann, sediment og biota revidert 30.10.2020 (M-nummer 608). (Quality standards for water, sediment and biota – revised 2020.10.30). In Norweign. https://www.miljodirektoratet.no/publikasjoner/2016/september-2016/grenseverdier-for-klassifisering-av-vann-sediment-og-biota/ (accessed 13 Aug 2021)

[CR47] Minelgaite G, van Alst N, Stephansen DA (2020). An exploratory study of benthic diatom communities in stormwater ponds of different land uses and varying biocide contamination. Aquat Ecol.

[CR48] Österlund H, Gelting J, Nordblad F (2012). Copper and nickel in ultrafiltered brackish water: Labile or non-labile?. Mar Chem.

[CR49] Rieuwerts JS, Thornton I, Farago ME, Ashmore MR (1998). Factors influencing metal bioavailability in soils: preliminary investigations for the development of a critical loads approach for metals. Chem Speciat Bioavailab.

[CR50] Sigg L, Black F, Buffle J (2006). Comparison of analytical techniques for dynamic trace metal speciation in natural freshwaters. Environ Sci Technol.

[CR51] Søberg LC, Vollertsen J, Blecken GT, Nielsen AH, Viklander M (2016). Bioaccumulation of heavy metals in two wet retention ponds. Urban Water J.

[CR52] Scholes L, Mensah R, Revitt DM, Jones RH (2007) An investigation of urban water and sediment ecotoxicity in relation to metal concentrations. In: Morrison G.M., Rauch S. (eds) Highway and urban environment. Alliance For Global Sustainability Bookseries, vol 12., pp 359–370. Springer, Dordrecht. 10.1007/978-1-4020-6010-6_32

[CR53] Starzec P, Lind BB, Lanngren A, Lindgren Å, Svenson T (2005). Technical and environmental functioning of detention ponds for the treatment of highway and road runoff. Water Air Soil Pollut.

[CR54] SEPA (Swedish Environmental Protection Agency). (2016). Generella riktvärden för förorenad mark (General guideline values for polluted soil). (In Swedish). Available at: https://www.naturvardsverket.se/om-oss/publikationer/5900/riktvarden-for-fororenad-mark/. Accessed 20 May 2022

[CR55] Tessier A, Campbell PGC, Bisson M (1979). Sequential extraction procedure for the speciation of particulate trace metals. Anal Chem.

[CR56] Tixier G, Rochfort Q, Grapentine L, Marsalek J, Lafont M (2012). Spatial and seasonal toxicity in a stormwater management facility: evidence obtained by adapting an integrated sediment quality assessment approach. Water Res.

[CR57] Uher E, Besse JP, Delaigue O, Husson F, Lebrun JD (2018). Comparison of the metal contamination in water measured by diffusive gradient in thin film (DGT), biomonitoring and total metal dissolved concentration at a national scale. Appl Geochemistry.

[CR58] Van Buren MA, Watt WE, Marsalek J (1996). Enhancing the removal of pollutants by on-stream pond. Wat Sci Tech.

[CR59] Van Leeuwen HP, Town RM, Buffle J, Cleven RFMJ, Davison W, Puy J, Van Riemsdijk WH, Sigg L (2005). Dynamic speciation analysis and bioavailability of metals in aquatic systems. Environ Sci Technol.

[CR60] Winston RJ, Hunt WF, Kennedy SG, Merriman LS, Chandler J, Brown D (2013). Evaluation of floating treatment wetlands as retrofits to existing stormwater retention ponds. Ecol Eng.

[CR61] Xie M, Simpson SL, Huang J, Teasdale PR, Wang WX (2021). In Situ DGT Sensing of bioavailable metal fluxes to improve toxicity predictions for sediments. Environ Sci Technol.

[CR62] Xu D, Gao B, Chen S et al (2019) Release risk assessment of trace metals in urban soils using in-situ DGT and DIFS model. Sci Total Environ 694694:133624.10.1016/j.scitotenv.2019.133624 10.1016/j.scitotenv.2019.133624 31401511

[CR63] Yousef YA, Hvitved-Jacobsen T, Harper HH, Lin LY (1990). Heavy metal accumulation and transport through detention ponds receiving highway runoff. Sci Total Environ.

[CR64] Zhang H, Davison W (2015). Use of diffusive gradients in thin-films for studies of chemical speciation and bioavailability. Environ Chem.

