# Correction to: Facilitating maintenance of stormwater ponds: comparison of analytical methods for determination of metal pollution

**DOI:** 10.1007/s11356-022-21428-y

**Published:** 2022-06-16

**Authors:** Snežana Gavrić, Kelsey Flanagan, Heléne Österlund, Godecke‑Tobias Blecken, Maria Viklander

**Affiliations:** Urban Water Engineering, Department of Civil, Environmental and Natural Resources Engineering, Luleå University of Technology, 971 87, Luleå, Sweden

**Correction to: Environmental Science and Pollution Research**

**https://doi.org/10.1007/s11356-022-20694-0**

The upper part of Fig. 6 should be removed as it was a track changes version of the image.

The Original article has been corrected.

