# Supplementary material for: Facilitating maintenance of stormwater ponds: comparison of analytical methods for determination of metal pollution
Source: Environ Sci Pollut Res Int. 2022 Jun 1;29(49):74877–93. doi: 10.1007/s11356-022-20694-0 (PMC9550750; doi:10.1007/s11356-022-20694-0)
Supplement: Supplementary file 1 — Supplementary file1 (DOCX 380 KB) [file 11356_2022_20694_MOESM1_ESM.docx]

Supplementary material for: **Facilitating maintenance of stormwater ponds: comparison of analytical methods for determination of metal pollution**

Snežana Gavrić*, Kelsey Flanagan, Heléne Österlund, Godecke-Tobias Blecken, and Maria Viklander

Urban Water Engineering, Department of Civil, Environmental and Natural Resources Engineering, Luleå University of Technology, 971 87 Luleå, Sweden

Corresponding author: snezana.gavric@ltu.se

Table S1: General limits of quantification (LOQ) for the different metal analysis. The quantification limits were increased for some samples due to extra dilution of the sample, which was needed to reduce matrix effects such as signal suppression.

| LOQ of different analysis | unit | Cd | Cr | Cu | Ni | Pb | Zn |
| --- | --- | --- | --- | --- | --- | --- | --- |
| Total metal concentrations | mg/kg DW | 0.1 | 0.25 | 0.1 | 1.0 | 0.5 | 1 |
| Pore water metal concentrations | µg/L | 0.002 | 0.01 | 0.1 | 0.05 | 0.01 | 0.2 |
| DGT labile concentrations* | µg | 0.0005 | 0.005 | 0.01 | 0.005 | 0.002 | 0.01 |
| Fraction 1 | mg/kg DW | 0.003 | 0.03 | 0.05 | 0.03 | 0.009 | 0.09 |
| Fraction 2 | mg/kg DW | 0.006 | 0.06 | 0.2 | 0.06 | 0.03 | 0.3 |
| Fraction 3 | mg/kg DW | 0.002 | 0.02 | 0.03 | 0.02 | 0.006 | 0.06 |
| Fraction 4 | mg/kg DW | 0.002 | 0.02 | 0.04 | 0.02 | 0.008 | 0.08 |
| Fraction 5 | mg/kg DW | 0.003 | 0.03 | 0.05 | 0.03 | 0.01 | 0.1 |

*reporting limits for the metal mass of an unused DGT

Table S2: Description of the five-step sequential extraction analysis

|  | Method | To remove and measure |
| --- | --- | --- |
| Fraction 1 | Extraction of 1 g sample with 10 ml 1.0 M acetate buffer (pH 5) by shaking for 6 h at room temperature | adsorbed and exchangeable metals and carbonates |
| Fraction 2 | Extraction of the solid residue from Step 1 with 50 ml 0.1 M pyrophosphate solution (pH 9) by shaking for 1 h at room temperature | labile organic forms |
| Fraction 3 | Extraction of the solid residue from Step 2 with 10 ml 0.25 M hydroxylamine hydrochloride for 4 h at 50°C | amorphous Fe/Mn oxides |
| Fraction 4 | Extraction of the solid residue from Step 3 with 15 ml 1 M hydroxylamine hydrochloride in 25% acetic acid for 3 h at 90°C | crystalline Fe oxides |
| Fraction 5 | adding 0.75 g potassium chlorate to the solid residue from Step 4 followed by adding 15 ml 12 M hydrochloric acid for 30 min at room temperature and then 10 ml 4 M nitric acid for 20 min at 90°C | stable organic forms and sulphides |

Table S3: Percent of censored data in 32 sediment samples for six metals in different analysis

| Method | Zn | Cu | Pd | Ni | Cr | Cd |
| --- | --- | --- | --- | --- | --- | --- |
| Total metal concentrations | 0 | 0 | 0 | 0 | 0 | 22 |
| Pore water metal concentrations | 0 | 16 | 6 | 0 | 0 | 16 |
| DGT labile concentrations | 0 | 0 | 0 | 0 | 3 | 6 |
| Fraction 1 | 0 | 0 | 0 | 0 | 0 | 0 |
| Fraction 2 | 3 | 16 | 6 | 44 | 31 | 84 |
| Fraction 3 | 0 | 0 | 0 | 0 | 0 | 0 |
| Fraction 4 | 0 | 0 | 0 | 0 | 0 | 0 |
| Fraction 5 | 0 | 0 | 0 | 0 | 0 | 3 |

**Guidelines included for the ranking of the total metal concentrations:**

The Swedish EPA guideline includes values derived for two different types of land use i.e., sensitive (S) and less sensitive (LS) land use (Swedish EPA, 2016). These guidelines were chosen for this study, as they were developed to consider human health risks due to direct and indirect contact with the contaminated soil as well as protection of the soil ecosystem, groundwater, and surface water (SEPA, 2016). Exceeding the less sensitive limit means that the soil quality limits the choice of land use to industries and roads and this limit is used for the ranking. Norwegian guidelines (Miljødirektoratet, 2016) include five classes: background concentration (I), low concentration (II), moderate concentration (III), high concentration (IV), and very high concentration (V) (Miljødirektoratet, 2016). The classification system serves to determine the environmental status in different water bodies (Olsen et al., 2019). The Norwegian EQS for contaminated sediments is generally lower compared to SEPA’s and was used for the assessment of ecotoxicological effects. For the ranking table, sediment samples that do not have good status (Class III-V) received a rank. The Canadian guideline includes two values i.e., interim sediment quality guidelines (ISQGs) and probable effect levels (PELs) that are used to assess the degree to which adverse biological effects are likely to occur due to exposure to metal levels in sediments (CCME, 2001). The values refer to total concentrations in upper sediment layer (5 cm) and define three ranges: i) concentrations below ISQGs, ii) concentrations between the ISQGS and PELs and iii) concentrations above PEL that are rarely, occasionally, and frequently associated with the adverse biological effects respectively (CCME, 2001).

**Method how the pore water and DGT concentrations are ranked based on the method described in HVMFS (2016):**

According to the HVMFS (2016), annual average dissolved metal concentration is compared to: i) the lower regulation limit (Table 2) to assign “good” status if there is no exceedance and ii) the upper regulation limit (Table 2) to assign “moderate” status (for Cu and Zn) and “not good” status (for Ni and Pb) if there is exceedance. If annual average dissolved concentrations fall inside the range (Table 2), the bioavailable concentrations are calculated using Bio-met and the annual average bioavailable concentrations are compared against the lower regulation limit (Table 2) to assign “moderate” status (for Cu and Zn) and “not good” status (for Ni and Pb) if there is exceedance. Where local physicochemical parameters are outside of the validated ranges for Bio-met (Table S4 in the Supplementary material), the tool automatically assigns the most appropriate validated value to calculate bioavailable concentration with a display of a warning message. For the samples were water chemistry falls outside of the validated range for Bio-met in addition to comparing bioavailable metal concentration to the regulatory value, measured dissolved metal concentrations are also compared to the “generic values” (Table S4 in the Supplementary material) and the worse scenario is used for the classification (HVMFS, 2016). For one sample (Or2-O), the DOC value was missing and the value from Or2-I is used instead, as it is recommended to use the value from the similar site in such cases (HVMFS, 2016). For the samples that had Cu below the LOQ (< 1 µg/L), comparison to the lower limit (0.5 µg/L) is not done but rather status is assigned as “good”.

Table S4: Validated water chemistry ranges for Cu, Ni and Zn in Bio-met (Bio-met, 2015) and percentage of samples with pH lower and DOC and Ca higher than the validated ranges.

| Parameter | Validation range Cu | [%] | Validation range Ni | [%] | Validation range Zn | [%] |
| --- | --- | --- | --- | --- | --- | --- |
| pH | 6.0-8.5 | 25 | 6.5-8.7 | 31 | 6.0-8.5 | 25 |
| DOC [mg/L] | 30 | 50 | 30 | 50 | 30 | 50 |
| Ca [mg/L] | 3.1-129 | 9 | 2-88* | 28 | 5.0-160^a^ | 3 |

^a^no increase in protective effect of calcium after this concentration (Bio-met, 2015)

Table S5: General parameters in 32 pond sediment samples

| Sample | pH | Cond | DO | DOC | Cl | TOC | LOI | N | C/N | Clay+silt | Sand |
| --- | --- | --- | --- | --- | --- | --- | --- | --- | --- | --- | --- |
|  |  | μS/cm | mg/L | mg/L | mg/L | % DW | % DW | mg/kg DW |  |  |  |
| Os1-I | 7.50 | 814 | 0.36 | 14.5 | 12 | 5.07 | 4.7 | 920 | 55.1 | 0.63 | 0.37 |
| Os1-O | 7.20 | 872 | 0.28 | 19.7 | 14.6 | 5.13 | 10.8 | 1600 | 32.1 | 0.86 | 0.14 |
| Or1-I | 7.8 | 471 | 0.4 | 10.2 | 84.8 | 0.29 | 1.5 | 620 | 4.68 | 0.80 | 0.20 |
| Or1-O | 6.7 | 645 | 0.163 | 24.2 | 98.9 | 0.6 | 2.1 | 1000 | 6.00 | 0.94 | 0.06 |
| Or2-I | 5.8 | 426 | 0.019 | 36.5 | 82.8 | 1.58 | 4.7 | 4800 | 3.29 | 1.00 | 0.00 |
| Or2-O | 5.9 | 618 | 0.101 | NA | NA | 1.86 | 4.1 | 3800 | 4.89 | 0.95 | 0.05 |
| Or3-I | 7.1 | 615 | 0.175 | 27.1 | 57.4 | 0.82 | 3.2 | 430 | 19.1 | 0.94 | 0.06 |
| Or3-O | 5.8 | 1481 | 0.233 | 31.9 | 51.5 | 1.86 | 5.8 | 2700 | 6.89 | 0.91 | 0.09 |
| Or4-I | 6.9 | 608 | 0.036 | 40.7 | 47.2 | 1.71 | 4.7 | 1500 | 11.4 | 0.81 | 0.19 |
| Or4-O | 6.5 | 53.8 | 0.262 | 47.7 | 47 | 1.42 | 6.5 | 1400 | 10.1 | 0.94 | 0.06 |
| Or5-I | 6.7 | 536 | 0.272 | 93.2 | 169 | 1.36 | 6.7 | 270 | 50.4 | 0.98 | 0.02 |
| Or5-O | 6.7 | 662 | 0.459 | 33.7 | 226 | 0.5 | 6.7 | 350 | 14.3 | 0.94 | 0.06 |
| Or6-I | 7.1 | 358 | 0.049 | 33.2 | 72.9 | 0.62 | 7.1 | 220 | 28.2 | 0.62 | 0.38 |
| Or6-O | 7.3 | 402 | 0.115 | 23 | 51 | 0.46 | 2.1 | 300 | 15.3 | 0.87 | 0.13 |
| S1-I | 7.7 | 623 | 0.02 | 15.7 | 71.6 | 10.1 | 17 | 3700 | 27.3 | 0.62 | 0.38 |
| S1-O | 7.9 | 566 | 0.02 | 7.99 | 79.2 | 13.4 | 20.6 | 5100 | 26.3 | 0.81 | 0.19 |
| S2-I | 7.5 | 465 | 0.02 | 25.7 | 39.5 | 2.2 | 5.3 | 1100 | 20.0 | 0.64 | 0.36 |
| S2-O | 6.6 | 618 | 0.027 | 57.2 | 48.3 | 6.43 | 12.6 | 4700 | 13.7 | 0.86 | 0.14 |
| S3-I | 6.9 | 637 | 0.006 | 33.1 | 164 | 19.8 | 17.4 | 3700 | 53.5 | 0.38 | 0.62 |
| S3-O | 6.7 | 474 | 0.011 | 50 | 149 | 22.1 | 35.9 | 8300 | 26.6 | 0.30 | 0.70 |
| S4-I | 7.6 | 3690 | 0.035 | 58.7 | 1510 | 9.34 | 13.6 | 2000 | 46.7 | 0.97 | 0.03 |
| S5-I | 6.9 | 540 | 4.19 | 124 | 144 | 3.28 | 6.4 | 930 | 35.3 | 0.22 | 0.72 |
| S5-O | 6.7 | 6180 | 0.021 | 57.6 | 3420 | 3.45 | 4.7 | 1300 | 26.5 | 0.55 | 0.45 |
| S6-I | 7.5 | 269 | 0.029 | 26.2 | 166 | 10.6 | 5.5 | 560 | 189 | 0.51 | 0.49 |
| S6-O | 7.4 | 816 | 0.03 | 40.2 | 267 | 3.62 | 7.4 | 1300 | 27.8 | 0.87 | 0.13 |
| V1-I | 6.1 | 777 | 0.035 | 23.2 | 275 | 10.2 | 11 | 1600 | 63.8 | 0.56 | 0.44 |
| V1-O | 5.9 | 207 | 0.005 | 24.2 | 35.5 | 8.62 | 4.3 | 1100 | 78.4 | 0.76 | 0.24 |
| V2-I | 6.0 | 255 | 0.026 | 18.6 | 58.1 | 5.36 | 7.3 | 1700 | 31.5 | 0.55 | 0.45 |
| V3-I | 5.7 | 177.2 | 0.001 | 10.6 | 50.5 | 7.64 | 13.2 | 2400 | 31.8 | 0.65 | 0.35 |
| V3-O | 5.9 | 371 | 0.037 | 9.6 | 85.4 | 14.2 | 23.8 | 16000 | 8.88 | 0.56 | 0.44 |
| V4-I | 5.1 | 637 | 0.001 | 51.7 | 156 | 9.99 | 16.4 | 3400 | 29.4 | 0.77 | 0.23 |
| V4-O | 5.8 | 825 | 0.001 | 127 | 198 | 8.45 | 14.9 | 3000 | 28.2 | 0.80 | 0.20 |

Table S6: Results of cendiff test. Test was run on total (T), pore water (PW) and DGT labile concentrations (DGT) for the two group of samples i.e., samples that had toxicity (EC20) reported and the rest.

| Concentrations | p-value |
| --- | --- |
| Zn_T_ | 0.451 |
| Cu_T_ | 0.125 |
| Pb_T_ | 0.053 |
| Ni_T_ | 0.231 |
| Cr_T_ | 0.094 |
| Cd_T_ | 0.480 |
|  |  |
| Zn_PW_ | 0.506 |
| Cu_PW_ | 0.801 |
| Pb_PW_ | 0.731 |
| Ni_PW_ | 0.702 |
| Cr_PW_ | 0.371 |
| Cd_PW_ | 0.775 |
|  |  |
| Zn_DGT_ | 0.221 |
| Cu_DGT_ | 0.403 |
| Pb_DGT_ | 0.535 |
| Ni_DGT_ | 0.033 |
| Cr_DGT_ | 0.664 |
| Cd_DGT_ | 0.707 |


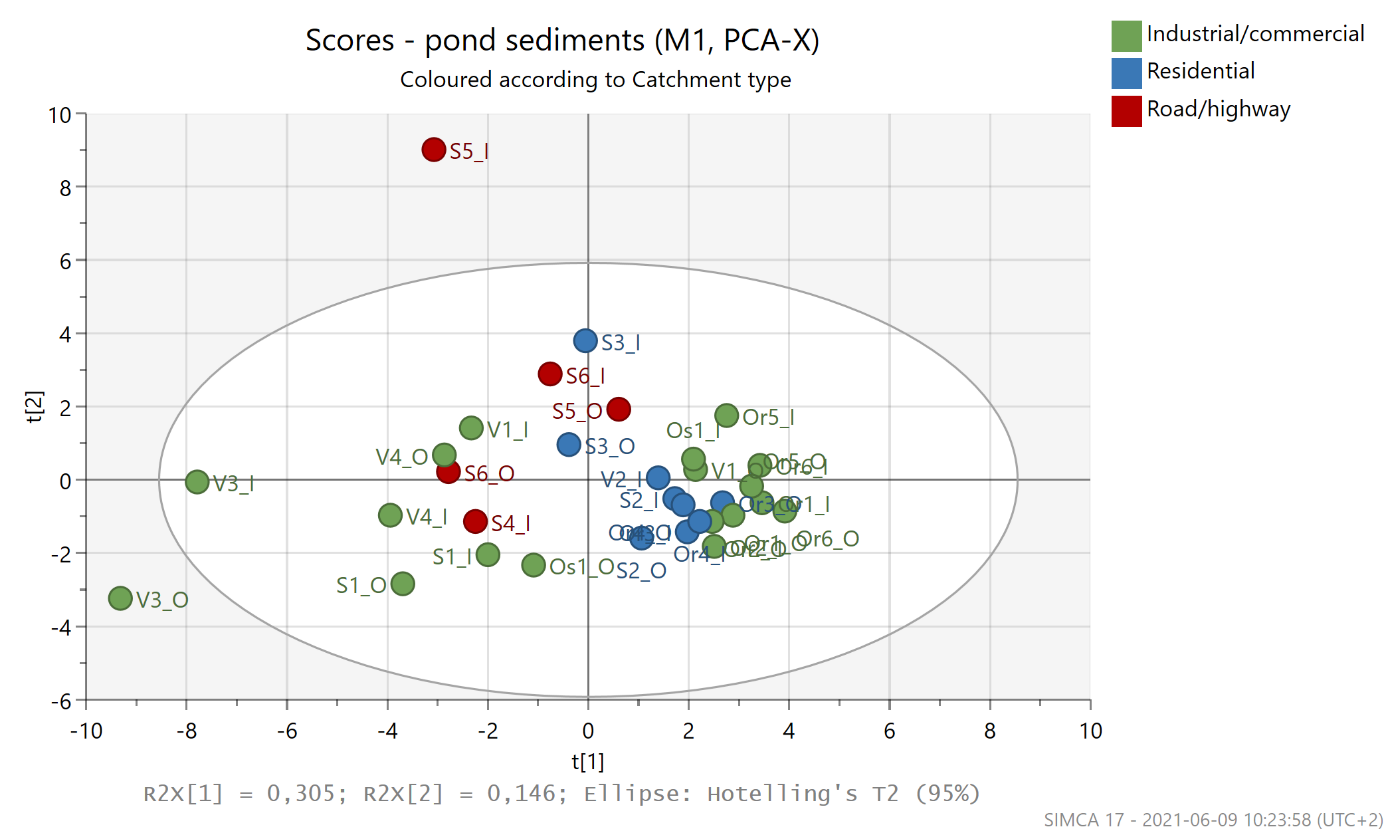


**Fig. S1**: Score plot for pond sediments where coloring indicates different catchment types and the labels sample names

Table S7: Results of Spearman rho correlation test between Fraction 1 of the sequential extraction (adsorbed and exchangeable metals and carbonates) and total metal concentration. Number of samples n=32.

| Metal | rho | p-value |
| --- | --- | --- |
| Zn | 0.92 | < 2.2E-16 |
| Cu | 0.74 | 1.33E-06 |
| Pb | 0.83 | 6.31E-09 |
| Ni | 0.58 | 5.21E-04 |
| Cr | 0.39 | 0.0275 |
| Cd | 0.696***** | 1.62E-08 |

* Kendall’s tau correlation (25% of the data was censored)


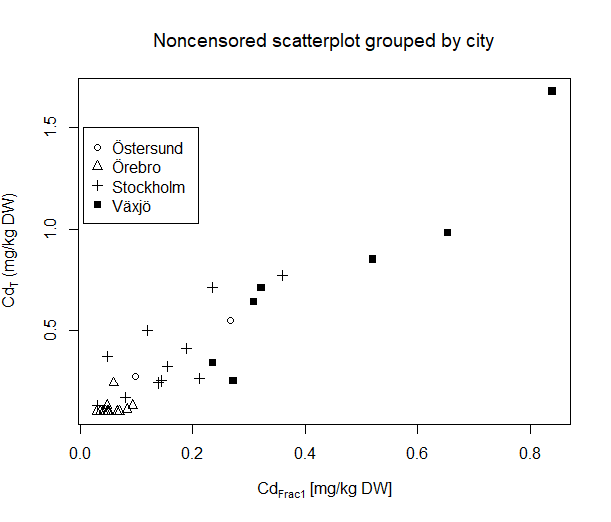

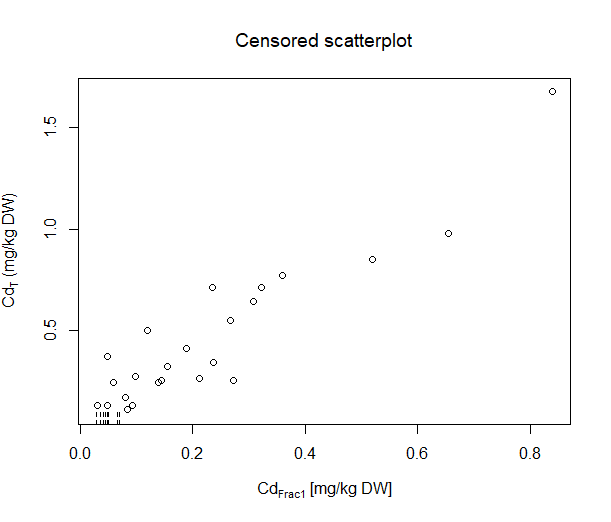


**Fig. S3**: Censored scatterplot shows total Cd concentration and Cd in Fraction 1 of sequential extraction

**Fig. S2**: Noncensored scatterplot shows total Cd concentration and Cd in Fraction 1 of sequential extraction where observations are grouped by city

Table S8: Correlation test results between total metal concentrations and different fractions of sequential extraction analysis. In case of censored values Kendall’s tau is calculated otherwise Spearman rho.

|  | **fcensored (%)** | **rho/tau** | **p-value** |
| --- | --- | --- | --- |
| **Zn_T_** | 0 |  |  |
| Fraction 1 | 0 | 0.92 | < 2.2e-16 |
| Fraction 2 | 3 | 0.718 | 8.05E-09 |
| Fraction 3 | 0 | 0.97 | < 2.2E-16 |
| Fraction 4 | 0 | 0.87 | 1.19E-10 |
| Fraction 5 | 0 | 0.46 | 0.007455 |
| **Cu_T_** | 0 |  |  |
| Fraction 1 | 0 | 0.74 | 1.33E-06 |
| Fraction 2 | 16 | 0.65 | 1.29E-07 |
| Fraction 3 | 0 | 0.92 | 4.48E-14 |
| Fraction 4 | 0 | 0.95 | < 2.2E-16 |
| Fraction 5 | 0 | 0.89 | 9.08E-12 |
| **Pb_T_** | 0 |  |  |
| Fraction 1 | 0 | 0.83 | 6.31E-09 |
| Fraction 2 | 6 | 0.631 | 4.07E-07 |
| Fraction 3 | 0 | 0.88 | 5.62E-11 |
| Fraction 4 | 0 | 0.72 | 3.04E-06 |
| Fraction 5 | 0 | 0.39 | 0.02554 |
| **Ni_T_** | 0 |  |  |
| Fraction 1 | 0 | 0.58 | 5.21E-04 |
| Fraction 2 | 44 | 0.204 | 0.082047 |
| Fraction 3 | 0 | 0.90 | 1.17E-12 |
| Fraction 4 | 0 | 0.71 | 4.77E-06 |
| Fraction 5 | 0 | 0.68 | 2.19E-05 |
| **Cr_T_** | 0 |  |  |
| Fraction 1 | 0 | 0.39 | 0.0275 |
| Fraction 2 | 31 | 0.274 | 0.023732 |
| Fraction 3 | 0 | 0.78 | 1.07E-07 |
| Fraction 4 | 0 | 0.75 | 9.92E-07 |
| Fraction 5 | 0 | 0.77 | 3.02E-07 |
| **Cd_T_** | 25 |  |  |
| Fraction 1 | 25 | 0.696 | 1.62E-08 |
| Fraction 2 | 84 | 0.083 | 0.46669 |
| Fraction 3 | 25 | 0.677 | 3.79E-08 |
| Fraction 4 | 25 | 0.637 | 2.30E-07 |
| Fraction 5 | 3 | 0.413 | 0.000795 |


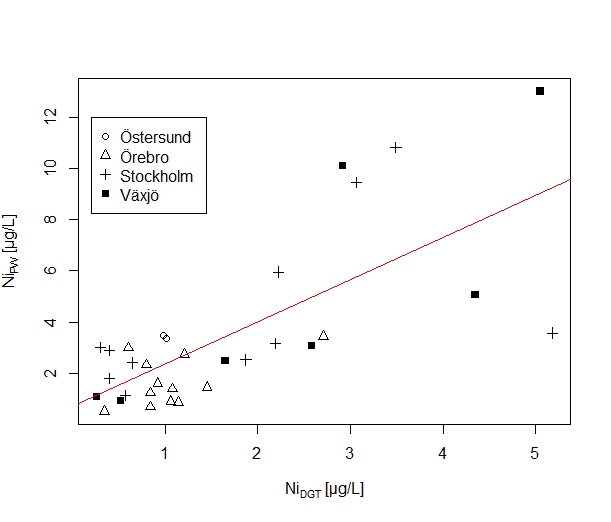


**Fig. S4**: All values were above LOQ. Noncensored scatterplot shows pore water Ni and DGT Ni concentration where observations are grouped by city


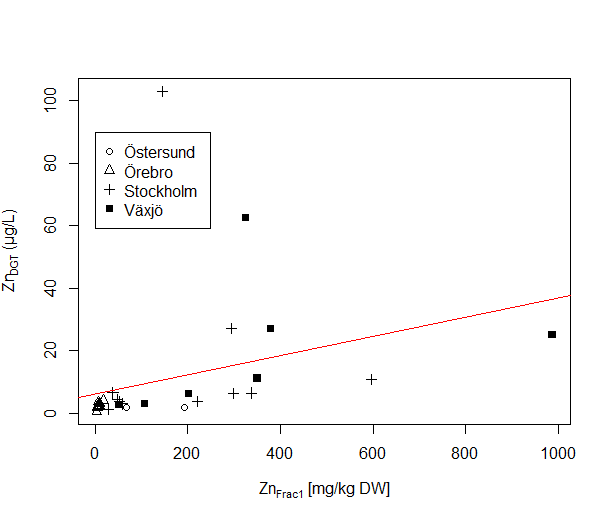


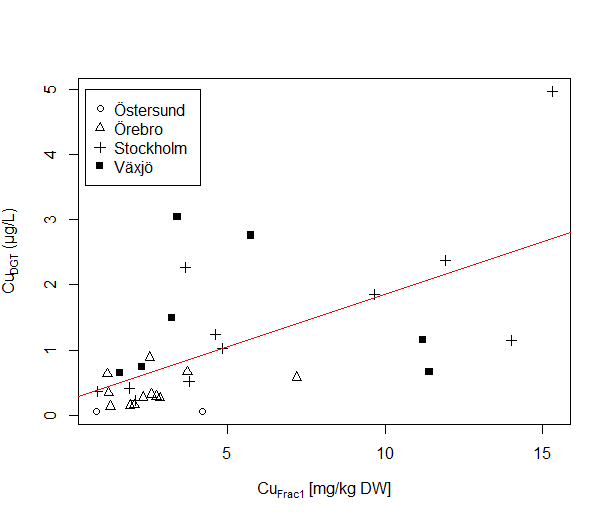


**Fig. S5**: All values were above LOQ. Noncensored scatterplot shows DGT Zn and Zn in Fraction 1 of sequential extraction (top graph), and DGT Cu and Cu in Fraction 1 of sequential extraction (bottom graph) where observations are grouped by city

Table S9: Correlation test results between LOI and total (T), pore water (PW) and DGT labile concentrations (DGT). In case of censored values Kendall’s tau is calculated otherwise Spearman rho.

| Metal analysis | f-censored [%] | p-value | tau or rho |
| --- | --- | --- | --- |
| Zn_T_ | 0 | 2.95E-04***** | 0.60 |
| Zn_PW_ | 0 | 0.096 | 0.30 |
| Zn_DGT_ | 0 | 0.028 | 0.39 |
| Cu_T_ | 0 | 4.83E-04***** | 0.58 |
| Cu_PW_ | 16▲ | 0.279 | 0.135 |
| Cu_DGT_ | 0 | 0.019 | 0.41 |
| Pb_T_ | 0 | 0.017 | 0.42 |
| Pb_PW_ | 6▲ | 0.337 | 0.121 |
| Pb_DGT_ | 0 | 0.109 | 0.29 |
| Ni_T_ | 0 | 0.071 | 0.32 |
| DNi_PW_ | 0 | 0.017 | 0.42 |
| Ni_DGT_ | 0 | 0.486 | 0.13 |
| Cr_T_ | 0 | 0.083 | 0.31 |
| Cr_PW_ | 0 | 4.73E-04***** | 0.58 |
| Cr_DGT_ | 3▲ | 0.961 | -0.008 |
| Cd_T_ | 25▲ | 1.42E-05***** | 0.534 |
| Cd_PW_ | 16▲ | 0.341 | 0.119 |
| Cd_DGT_ | 6▲ | 0.059 | 0.236 |

▲indicates % of censored data and that Kendall’s tau is calculated

* indicates statistical significance (p<0.01)

Table S10: Kendall’s tau correlation coefficient between TOC and Fraction 2 of sequential extraction analysis (labile organic forms).

| Metal analysis | f-censored [%] | p-value | tau |
| --- | --- | --- | --- |
| Zn_Frac2_ | 3 | 7.31E-06* | 0.558 |
| Cu_Frac2_ | 16 | 3.09E-04* | 0.446 |
| Pb_Frac2_ | 6 | 8.24E-04* | 0.417 |
| Ni_Frac2_ | 44 | 0.025 | 0.262 |
| Cr_Frac2_ | 31 | 5.96E-04* | 0.415 |
| Cd_Frac2_ | 84 | 0.397 | 0.097 |

* indicates statistical significance (p<0.01)


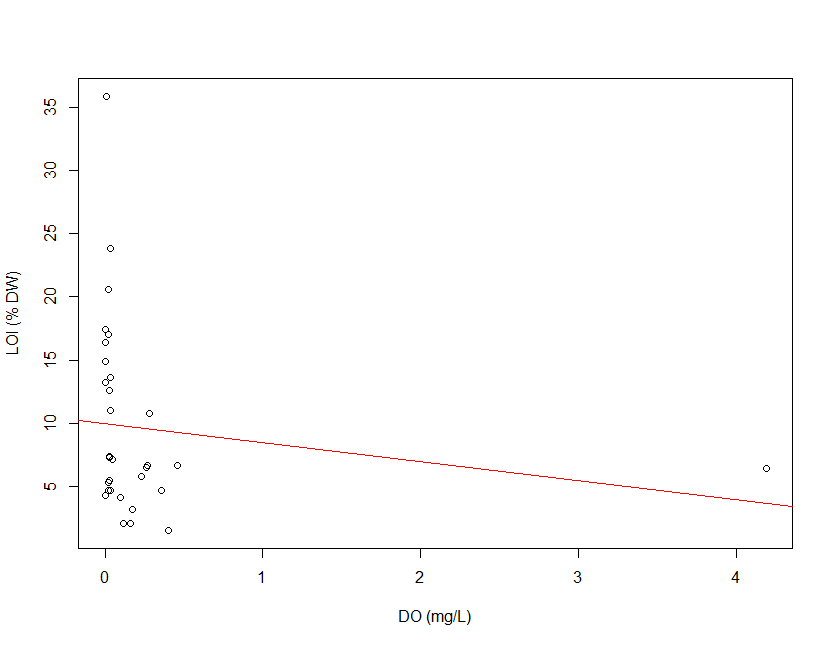

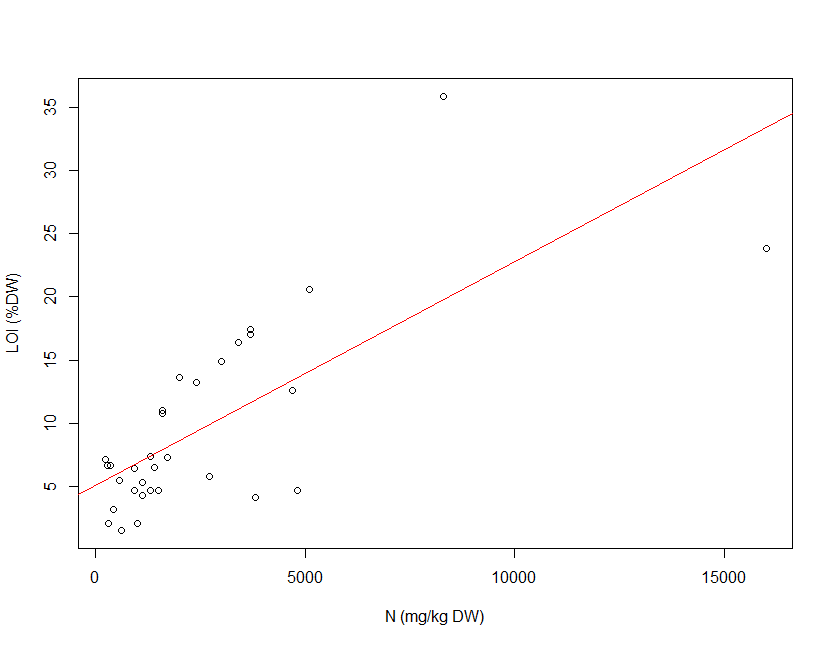

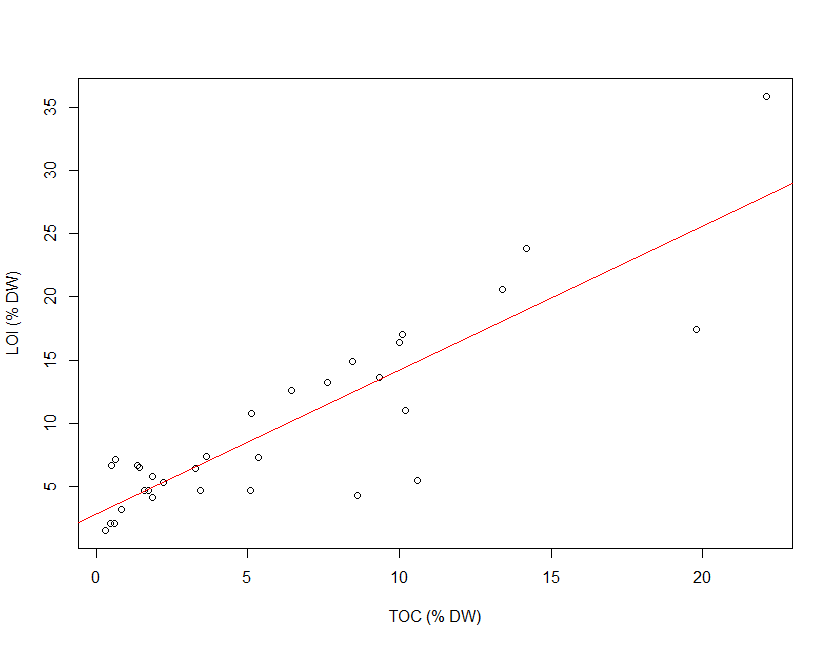


**Fig. S6**: Scatterplots between LOI and TOC (ρ=0.76, p= 4.46E-07), LOI and N (ρ=0.618, p=1.62E-04), and LOI and DO (ρ=-0.46, p= 0.008)


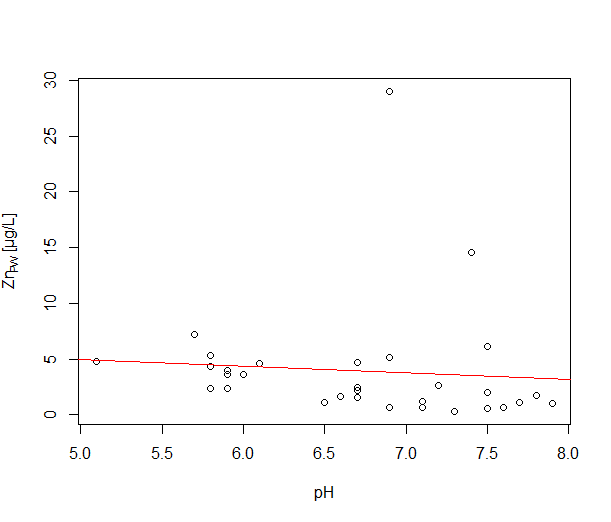


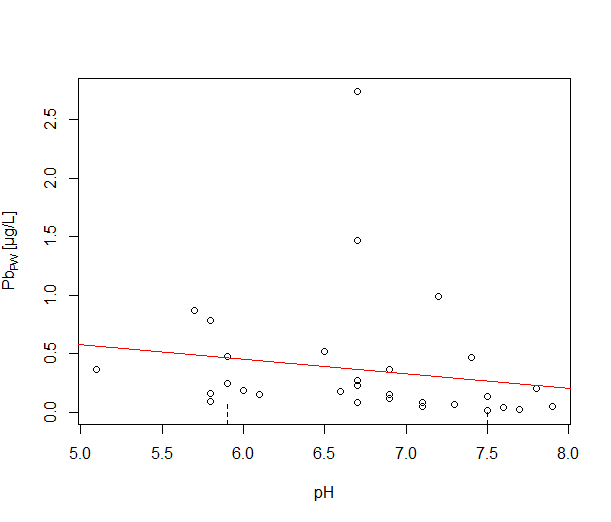


**Fig. S7**: Scatterplots between pH and pore water Zn concentration (ρ=-0.466, p= 0.007) and pH and pore water Pb concentration (tau=-0.337, p= 0.007)

Table S11: Total metal concentrations in stormwater pond sediments

|  | Total Cd | Total Cr | Total Cu | Total Ni | Total Pb | Total Zn |
| --- | --- | --- | --- | --- | --- | --- |
| Os1_In | 0.27 | 14.6 | 39.2 | 17.2 | 14.6 | 218 |
| Os1_Out | 0.55 | 23.9 | 114 | 23.7 | 91.8 | 506 |
| Or1_In | <0.1 | 19.8 | 18 | 11.5 | 15.9 | 51.2 |
| Or1_Out | <0.1 | 23.2 | 26.1 | 13.4 | 18 | 75.2 |
| Or2_In | 0.13 | 37.4 | 26.9 | 24.9 | 18.6 | 92 |
| Or2_Out | <0.1 | 37.1 | 28.5 | 24.2 | 18 | 87.7 |
| Or3_In | 0.24 | 46.8 | 43.7 | 30.6 | 25 | 102 |
| Or3_Out | <0.1 | 22.9 | 19.4 | 19.4 | 12.4 | 59.4 |
| Or4_In | 0.13 | 38.4 | 29.7 | 27.9 | 19.6 | 87.2 |
| Or4_Out | 0.11 | 35.1 | 29.7 | 25.1 | 18.7 | 76.4 |
| Or5_In | <0.1 | 16.8 | 14.5 | 9.3 | 15.1 | 51.8 |
| Or5_Out | <0.1 | 14.4 | 13.4 | 8.8 | 13.2 | 43.6 |
| Or6_In | <0.1 | 8.44 | 6.14 | 3.7 | 8.8 | 27.4 |
| Or6_Out | <0.1 | 13.3 | 8.18 | 6.9 | 12.7 | 31.8 |
| S1_In | 0.71 | 54.8 | 253 | 21.3 | 47.3 | 680 |
| S1_Out | 0.77 | 71.9 | 319 | 27.2 | 54.4 | 886 |
| S2_In | 0.24 | 26.2 | 36.1 | 17.9 | 17.4 | 129 |
| S2_Out | 0.26 | 40.2 | 55.4 | 29.4 | 19.2 | 156 |
| S3_In | 0.5 | 15.2 | 23.4 | 11.6 | 9 | 247 |
| S3_Out | 0.41 | 18.4 | 32.8 | 15.5 | 9.4 | 69.8 |
| S4_In | 0.32 | 58.2 | 172 | 24.9 | 35.3 | 477 |
| S5_In | 0.37 | 28.7 | 63.6 | 12.3 | 13.4 | 255 |
| S5_Out | 0.17 | 36.7 | 41.6 | 19.8 | 20.3 | 104 |
| S6_In | 0.13 | 28.5 | 60.9 | 10.2 | 9.5 | 330 |
| S6_Out | 0.25 | 54.2 | 210 | 22.9 | 30.2 | 1020 |
| V1_In | 0.64 | 21.3 | 93 | 10.3 | 37.7 | 643 |
| V1_Out | 0.25 | 8.44 | 10.9 | 3.4 | 7 | 122 |
| V2_In | 0.34 | 13.6 | 33.3 | 6.7 | 29.6 | 127 |
| V3_In | 0.98 | 34.6 | 104 | 28.7 | 63 | 603 |
| V3_Out | 1.68 | 38.4 | 182 | 41.9 | 65.8 | 1380 |
| V4_In | 0.85 | 43.4 | 99.9 | 43.6 | 51.5 | 726 |
| V4_Out | 0.71 | 45 | 74.9 | 34.7 | 49.1 | 468 |

Table S12: Porewater metal concentrations in stormwater pond sediments

|  | Cd porewater | Cr porewater | Cu porewater | Ni porewater | Pb porewater | Zn porewater |
| --- | --- | --- | --- | --- | --- | --- |
| Os1_In | 0.02 | 0.367 | 0.302 | 3.46 | 0.138 | 2.01 |
| Os1_Out | 0.017 | 0.291 | 0.643 | 3.34 | 0.992 | 2.62 |
| Or1_In | 0.0049 | 0.0482 | 0.407 | 0.681 | 0.204 | 1.71 |
| Or1_Out | 0.00424 | 0.327 | 0.382 | 0.864 | 0.232 | 2.19 |
| Or2_In | 0.00318 | 0.951 | 0.299 | 0.897 | 0.0946 | 2.38 |
| Or2_Out | 0.00954 | 0.705 | 1.09 | 2.98 | 0.478 | 3.97 |
| Or3_In | 0.00319 | 0.357 | 1.82 | 1.61 | 0.087 | 0.685 |
| Or3_Out | 0.00624 | 0.921 | 1.25 | 2.32 | 0.782 | 4.37 |
| Or4_In | 0.00436 | 0.601 | 0.769 | 1.39 | 0.117 | 0.702 |
| Or4_Out | 0.00347 | 0.99 | 3.08 | 3.44 | 0.519 | 1.11 |
| Or5_In | 0.0249 | 2.71 | 3.55 | 2.74 | 2.74 | 4.71 |
| Or5_Out | 0.00538 | 1.54 | 0.947 | 1.44 | 1.47 | 1.57 |
| Or6_In | <0.002 | 0.292 | 0.626 | 1.24 | 0.0522 | 1.24 |
| Or6_Out | <0.002 | 0.121 | 0.197 | 0.514 | 0.0656 | 0.318 |
| S1_In | 0.00339 | 1.93 | 0.131 | 1.8 | 0.0256 | 1.09 |
| S1_Out | 0.00766 | 1.55 | 0.382 | 1.14 | 0.0497 | 1.03 |
| S2_In | 0.00328 | 0.359 | <0.1 | 2.52 | <0.01 | 0.542 |
| S2_Out | 0.0041 | 0.592 | 0.504 | 2.4 | 0.175 | 1.63 |
| S3_In | 0.0317 | 4.72 | 7.4 | 2.88 | 0.371 | 29 |
| S3_Out | 0.00589 | 4.42 | 2.03 | 3.02 | 0.272 | 2.42 |
| S4_In | 0.00506 | 2.77 | 0.309 | 3.57 | 0.0441 | 0.623 |
| S5_In | 0.0383 | 5.58 | 1.6 | 10.8 | 0.151 | 5.15 |
| S5_Out | 0.021 | 5.98 | <0.5 | 3.16 | 0.0883 | 2.41 |
| S6_In | 0.00984 | 3.59 | 0.324 | 9.45 | 0.0133 | 6.12 |
| S6_Out | 0.0212 | 4.39 | 3.36 | 5.95 | 0.468 | 14.6 |
| V1_In | <0.02 | 0.517 | <1 | 2.47 | 0.152 | 4.58 |
| V1_Out | <0.02 | 0.487 | <1 | 1.08 | <0.1 | 2.38 |
| V2_In | 0.0047 | 0.524 | 0.535 | 0.917 | 0.188 | 3.64 |
| V3_In | 0.0129 | 3.78 | 1.38 | 13 | 0.872 | 7.17 |
| V3_Out | 0.005 | 3.07 | 0.779 | 3.06 | 0.245 | 3.58 |
| V4_In | 0.00883 | 7.12 | 0.667 | 5.07 | 0.363 | 4.81 |
| V4_Out | <0.02 | 11.1 | <1 | 10.1 | 0.164 | 5.32 |

Table S13: DGT metal concentrations in stormwater pond sediments

|  | Cd DGT | Cr DGT | Cu DGT | Ni  DGT | Pb DGT | Zn DGT |
| --- | --- | --- | --- | --- | --- | --- |
| Os1_In | <0.002 | 0.195 | 0.0598 | 0.978 | 0.0186 | 1.86 |
| Os1_Out | <0.002 | <0.191 | 0.0592 | 1.01 | 0.0401 | 1.84 |
| Or1_In | 0.00258 | 0.562 | 0.289 | 0.838 | 0.056 | 1.95 |
| Or1_Out | 0.00302 | 0.479 | 0.667 | 1.14 | 0.204 | 4.15 |
| Or2_In | 0.00223 | 0.417 | 0.132 | 1.06 | 0.0373 | 2.03 |
| Or2_Out | 0.00094 | 0.492 | 0.161 | 0.605 | 0.0338 | 2.5 |
| Or3_In | 0.00635 | 0.536 | 0.58 | 0.92 | 0.173 | 2.2 |
| Or3_Out | 0.00158 | 0.477 | 0.338 | 0.798 | 0.051 | 2.35 |
| Or4_In | 0.00331 | 0.561 | 0.319 | 1.08 | 0.0605 | 2.41 |
| Or4_Out | 0.0113 | 0.609 | 0.261 | 2.71 | 0.0323 | 3.62 |
| Or5_In | 0.0104 | 0.641 | 0.886 | 1.21 | 0.124 | 3.16 |
| Or5_Out | 0.0109 | 0.671 | 0.27 | 1.45 | 0.0583 | 3.39 |
| Or6_In | 0.00406 | 0.704 | 0.629 | 0.84 | 0.0836 | 2.51 |
| Or6_Out | 0.00174 | 0.544 | 0.147 | 0.342 | 0.103 | 0.688 |
| S1_In | 0.00441 | 0.504 | 1.02 | 0.396 | 0.0669 | 3.87 |
| S1_Out | 0.00727 | 0.552 | 2.37 | 0.573 | 0.0608 | 6.53 |
| S2_In | 0.00312 | 0.497 | 0.511 | 1.87 | 0.0901 | 4.32 |
| S2_Out | 0.0023 | 0.473 | 0.226 | 0.643 | 0.0431 | 3.03 |
| S3_In | 0.00529 | 0.451 | 0.416 | 0.4 | 0.0991 | 3.83 |
| S3_Out | 0.00183 | 0.297 | 0.358 | 0.296 | 0.0361 | 1.36 |
| S4_In | 0.00281 | 0.558 | 1.15 | 5.18 | 0.113 | 6.29 |
| S5_In | 0.0407 | 0.393 | 4.97 | 3.49 | 0.0496 | 103 |
| S5_Out | 0.00595 | 0.425 | 1.24 | 2.19 | 0.0498 | 6.56 |
| S6_In | 0.00319 | 0.406 | 2.27 | 3.06 | 0.156 | 27.3 |
| S6_Out | 0.00512 | 0.522 | 1.85 | 2.22 | 0.25 | 10.8 |
| V1_In | 0.0621 | 0.363 | 1.49 | 1.65 | 0.315 | 62.5 |
| V1_Out | 0.00548 | 0.333 | 0.648 | 0.261 | 0.0153 | 3.08 |
| V2_In | 0.00826 | 0.33 | 0.741 | 0.525 | 0.125 | 2.73 |
| V3_In | 0.0453 | 0.513 | 3.04 | 5.05 | 1.06 | 26.9 |
| V3_Out | 0.0245 | 0.391 | 2.76 | 2.58 | 0.413 | 25 |
| V4_In | 0.00901 | 0.713 | 1.15 | 4.35 | 0.264 | 11.2 |
| V4_Out | 0.0114 | 0.856 | 0.668 | 2.92 | 0.162 | 6.21 |

Table S14: Metal concentrations in Fraction 1 of sequential extraction analysis

|  | Cd  Frac 1 | Cr  Frac 1 | Cu  Frac 1 | Ni  Frac 1 | Pb  Frac 1 | Zn  Frac 1 |
| --- | --- | --- | --- | --- | --- | --- |
| Os1_In | 0.0973 | 1 | 0.859 | 1.88 | 7.33 | 66.3 |
| Os1_Out | 0.266 | 2.35 | 4.19 | 4.28 | 59.2 | 193 |
| Or1_In | 0.048 | 0.484 | 2.75 | 1.2 | 6.02 | 4.8 |
| Or1_Out | 0.0652 | 0.441 | 3.72 | 1.71 | 6.43 | 18.1 |
| Or2_In | 0.0475 | 0.537 | 1.28 | 2.24 | 4.5 | 8.54 |
| Or2_Out | 0.07 | 0.613 | 2.06 | 2.28 | 3.81 | 9.16 |
| Or3_In | 0.0595 | 0.525 | 7.2 | 1.43 | 8.15 | 5.46 |
| Or3_Out | 0.044 | 1.02 | 1.24 | 3.78 | 4.32 | 6.28 |
| Or4_In | 0.0934 | 0.516 | 2.59 | 3.7 | 5.82 | 5.86 |
| Or4_Out | 0.0829 | 1.08 | 2.86 | 4.65 | 6.08 | 6.78 |
| Or5_In | 0.0413 | 0.583 | 2.54 | 0.786 | 3.25 | 9.3 |
| Or5_Out | 0.0505 | 0.658 | 2.32 | 1.42 | 3.58 | 8.36 |
| Or6_In | 0.035 | 0.738 | 1.2 | 0.656 | 2.55 | 8.49 |
| Or6_Out | 0.0294 | 0.51 | 1.91 | 0.806 | 3.84 | 3.54 |
| S1_In | 0.235 | 4.89 | 4.85 | 5.72 | 12.7 | 220 |
| S1_Out | 0.359 | 4.68 | 11.9 | 5.04 | 18.4 | 337 |
| S2_In | 0.139 | 1.32 | 3.78 | 4.13 | 8.71 | 48.7 |
| S2_Out | 0.211 | 1.43 | 2.07 | 5.05 | 7.2 | 59.2 |
| S3_In | 0.119 | 1.33 | 1.89 | 2.91 | 2.21 | 52.2 |
| S3_Out | 0.189 | 2.7 | 0.876 | 3.36 | 2.62 | 27.8 |
| S4_In | 0.155 | 5.99 | 14 | 6.57 | 14.9 | 299 |
| S5_In | 0.0475 | 2.69 | 15.3 | 3.97 | 3.16 | 145 |
| S5_Out | 0.0808 | 2 | 4.63 | 3.4 | 5.73 | 38.2 |
| S6_In | 0.0299 | 4.14 | 3.66 | 4.59 | 5.56 | 294 |
| S6_Out | 0.144 | 5.28 | 9.67 | 5.48 | 15.3 | 597 |
| V1_In | 0.308 | 2.87 | 3.25 | 4.25 | 13.5 | 326 |
| V1_Out | 0.272 | 3.05 | 1.59 | 2.82 | 0.901 | 107 |
| V2_In | 0.236 | 2.74 | 2.29 | 2.8 | 4.4 | 50.6 |
| V3_In | 0.654 | 9.75 | 3.41 | 19.9 | 34.3 | 379 |
| V3_Out | 0.839 | 6.24 | 5.74 | 19.1 | 29.4 | 987 |
| V4_In | 0.52 | 2.78 | 11.2 | 4.54 | 10.1 | 350 |
| V4_Out | 0.322 | 3.23 | 11.4 | 3.79 | 4.24 | 203 |

Table S15: Metal concentrations in Fraction 2 of sequential extraction analysis

|  | Cd  Frac 2 | Cr  Frac 2 | Cu  Frac 2 | Ni  Frac 2 | Pb  Frac 2 | Zn  Frac 2 |
| --- | --- | --- | --- | --- | --- | --- |
| Os1_In | <0.3 | <3 | <5 | <3 | <1 | <11 |
| Os1_Out | <0.3 | 2.91 | 6.19 | <3 | 7.02 | 25.2 |
| Or1_In | <0.01 | 0.319 | 1.96 | 0.36 | 1.01 | 1.62 |
| Or1_Out | <0.005 | 0.301 | 1.99 | 0.425 | 1.39 | 3.2 |
| Or2_In | <0.006 | 0.633 | 1.94 | 0.604 | 2.01 | 2.3 |
| Or2_Out | 0.00813 | 0.609 | 4.08 | 0.549 | 1.68 | 4.05 |
| Or3_In | <0.006 | 0.556 | 5.51 | 0.364 | 2.71 | 3.15 |
| Or3_Out | <0.005 | 0.821 | 3.24 | 1.16 | 1.81 | 2.08 |
| Or4_In | 0.00922 | 0.491 | 5.13 | 1.03 | 2.48 | 3.14 |
| Or4_Out | <0.005 | 0.607 | 3.67 | 0.833 | 1.63 | 1.86 |
| Or5_In | <0.006 | 0.5 | 1.61 | 0.266 | 1.11 | 2.36 |
| Or5_Out | <0.006 | 0.556 | 2.05 | 0.41 | 1.13 | 2.22 |
| Or6_In | <0.006 | 0.431 | 1.12 | 0.215 | 0.632 | 1.2 |
| Or6_Out | <0.006 | 0.36 | 1.21 | 0.294 | 0.712 | 5.08 |
| S1_In | <0.3 | 3.62 | 11 | <3 | 7.02 | 28 |
| S1_Out | <0.3 | 4.81 | 14.4 | <3 | 11.1 | 57.5 |
| S2_In | 0.00791 | 0.879 | 4.02 | 0.609 | 1.54 | 12.1 |
| S2_Out | 0.0419 | 1.01 | 8.46 | 1.78 | 4.18 | 18.9 |
| S3_In | <0.1 | <1 | 3.75 | 1.75 | 1.18 | 9.93 |
| S3_Out | <0.1 | 1.76 | 5.61 | 3.56 | 3.12 | 14.8 |
| S4_In | <0.3 | <3 | 12.7 | <3 | 5.21 | 43.8 |
| S5_In | <0.1 | <1 | 7.18 | <1 | 0.8 | 18.5 |
| S5_Out | <0.1 | <1 | 5.14 | <1 | 1.5 | 6.65 |
| S6_In | <0.3 | <3 | <5 | <3 | <1 | 20.2 |
| S6_Out | <0.3 | <3 | 9.67 | <3 | 3.08 | 69.6 |
| V1_In | <0.3 | <3 | <6 | <3 | 3.83 | 45.6 |
| V1_Out | <0.3 | <3 | <6 | <3 | 2.75 | 26.7 |
| V2_In | <0.3 | <3 | <6 | <3 | 1.59 | 14 |
| V3_In | <0.3 | 4.71 | 8.69 | 4.19 | 12.2 | 59.4 |
| V3_Out | 0.221 | 6.85 | 16.5 | 5.47 | 14.8 | 242 |
| V4_In | <0.3 | 4.1 | 13.7 | <3 | 5.69 | 75.7 |
| V4_Out | <0.3 | 4.55 | 12.2 | <3 | 7.08 | 54.6 |

Table S16: Metal concentrations in Fraction 3 of sequential extraction analysis

|  | Cd  Frac 3 | Cr  Frac 3 | Cu  Frac 3 | Ni  Frac 3 | Pb  Frac 3 | Zn  Frac 3 |
| --- | --- | --- | --- | --- | --- | --- |
| Os1_In | 0.0675 | 2.56 | 15.9 | 2.96 | 3.99 | 51.7 |
| Os1_Out | 0.181 | 4.8 | 51 | 5.51 | 23.8 | 149 |
| Or1_In | 0.024 | 2.07 | 5.02 | 2.63 | 4.4 | 14.8 |
| Or1_Out | 0.0403 | 2.38 | 8.52 | 3.04 | 6.42 | 22.8 |
| Or2_In | 0.0236 | 3.21 | 6.7 | 5.12 | 6.52 | 25.4 |
| Or2_Out | 0.0353 | 3.53 | 4.9 | 5.8 | 7.29 | 28.6 |
| Or3_In | 0.0617 | 3.76 | 7.43 | 6.71 | 5.46 | 23.8 |
| Or3_Out | 0.0174 | 3.68 | 4.94 | 4.44 | 4.12 | 21.3 |
| Or4_In | 0.0343 | 4.05 | 5.32 | 6.83 | 6.53 | 27.4 |
| Or4_Out | 0.0238 | 4.12 | 7.04 | 5.77 | 6.33 | 26.2 |
| Or5_In | 0.0248 | 1.54 | 2.93 | 1.8 | 3.46 | 13.5 |
| Or5_Out | 0.0313 | 1.52 | 2.78 | 1.95 | 3.88 | 12.1 |
| Or6_In | 0.00717 | 0.975 | 1.23 | 0.651 | 2.76 | 7.28 |
| Or6_Out | 0.0188 | 1.84 | 1.86 | 2.03 | 3.76 | 13.1 |
| S1_In | 0.162 | 8.88 | 56.5 | 5 | 10.6 | 93.6 |
| S1_Out | 0.158 | 7.81 | 68.1 | 4.2 | 9.85 | 176 |
| S2_In | 0.0548 | 3.66 | 12.3 | 4.46 | 5.83 | 41.7 |
| S2_Out | 0.0755 | 4.3 | 7.88 | 7.97 | 7.43 | 47.2 |
| S3_In | 0.0577 | 2.65 | 3.26 | 4.72 | 2.09 | 40 |
| S3_Out | 0.143 | 3.32 | 2.73 | 6.52 | 3.26 | 25.6 |
| S4_In | 0.0608 | 9.47 | 58.4 | 5.3 | 12.3 | 172 |
| S5_In | 0.0209 | 7.21 | 22.7 | 3.78 | 4.86 | 107 |
| S5_Out | 0.0354 | 3.9 | 12.4 | 3.57 | 6.24 | 34.9 |
| S6_In | 0.0246 | 6.23 | 56.5 | 2.89 | 4.1 | 106 |
| S6_Out | 0.0837 | 9.42 | 106 | 5.38 | 11.9 | 363 |
| V1_In | 0.263 | 7.14 | 49.1 | 4.4 | 15.7 | 222 |
| V1_Out | 0.0775 | 2.32 | 5.03 | 1.51 | 3.93 | 36.9 |
| V2_In | 0.0876 | 3.62 | 16.4 | 2.54 | 3.8 | 49.7 |
| V3_In | 0.344 | 8.22 | 57.6 | 10 | 20.9 | 198 |
| V3_Out | 0.825 | 8.88 | 106 | 14.1 | 25.9 | 408 |
| V4_In | 0.202 | 7.71 | 39.9 | 14.7 | 26.4 | 271 |
| V4_Out | 0.212 | 6.2 | 26.9 | 12.7 | 29.8 | 206 |

Table S17: Metal concentrations in Fraction 4 of sequential extraction analysis

|  | Cd  Frac 4 | Cr  Frac 4 | Cu  Frac 4 | Ni  Frac 4 | Pb  Frac 4 | Zn  Frac 4 |
| --- | --- | --- | --- | --- | --- | --- |
| Os1_In | 0.0314 | 6.18 | 8.49 | 7.48 | 3.78 | 33.3 |
| Os1_Out | 0.0708 | 13.5 | 47.4 | 12.9 | 7.15 | 96 |
| Or1_In | 0.0122 | 3.34 | 2.64 | 2.45 | 2.22 | 11.7 |
| Or1_Out | 0.0138 | 5.72 | 4.28 | 4.16 | 3.06 | 15.8 |
| Or2_In | 0.0156 | 8.45 | 6.87 | 7.14 | 3.38 | 23.3 |
| Or2_Out | 0.0139 | 8.41 | 2.18 | 5.78 | 4.02 | 22.9 |
| Or3_In | 0.0325 | 7.24 | 4.89 | 5.13 | 2.28 | 19 |
| Or3_Out | 0.00741 | 5.41 | 3.45 | 3.71 | 1.9 | 15.2 |
| Or4_In | 0.0111 | 7.33 | 3.03 | 5.52 | 2.33 | 38.8 |
| Or4_Out | 0.011 | 7.46 | 4.51 | 5.34 | 3.09 | 19.9 |
| Or5_In | 0.0119 | 3.33 | 2.08 | 1.98 | 1.69 | 10.3 |
| Or5_Out | 0.015 | 3.1 | 1.97 | 2.07 | 1.62 | 10.7 |
| Or6_In | 0.00987 | 2.52 | 1.32 | 1.53 | 2.32 | 7.14 |
| Or6_Out | 0.0111 | 3.52 | 1.57 | 1.94 | 2.79 | 8.3 |
| S1_In | 0.0439 | 11.7 | 38.7 | 3.53 | 2.99 | 36.3 |
| S1_Out | 0.0821 | 17 | 99.9 | 5.6 | 6.93 | 93.5 |
| S2_In | 0.0198 | 8.49 | 10.4 | 5.56 | 2.53 | 23.5 |
| S2_Out | 0.0171 | 10.4 | 13.2 | 7.6 | 3.18 | 34.8 |
| S3_In | 0.0147 | 3.49 | 5.98 | 3.33 | 1.1 | 15.8 |
| S3_Out | 0.0254 | 5.49 | 13.8 | 5.14 | 1.81 | 19.3 |
| S4_In | 0.0371 | 18.2 | 60 | 7.27 | 6.71 | 67.9 |
| S5_In | 0.0142 | 14.9 | 13.3 | 4.17 | 2.81 | 37 |
| S5_Out | 0.0208 | 13.4 | 13.9 | 6.64 | 5.62 | 30.7 |
| S6_In | 0.0195 | 16.7 | 16.7 | 4.64 | 3.87 | 30.2 |
| S6_Out | 0.0453 | 17.9 | 74 | 6.47 | 4.84 | 79.8 |
| V1_In | 0.0472 | 14.9 | 41.7 | 6.59 | 6.31 | 59.9 |
| V1_Out | 0.0139 | 2.93 | 2.19 | 1.62 | 0.961 | 15 |
| V2_In | 0.0218 | 4.59 | 6.98 | 2.25 | 1.63 | 22.5 |
| V3_In | 0.0633 | 10.6 | 34.1 | 4.4 | 4.32 | 37.7 |
| V3_Out | 0.054 | 11.8 | 46.1 | 5.36 | 3.97 | 57.2 |
| V4_In | 0.0543 | 12.6 | 30.2 | 22 | 13.4 | 78 |
| V4_Out | 0.0436 | 12.9 | 19.9 | 18.7 | 12.3 | 73.5 |

Table S18: Metal concentrations in Fraction 5 of sequential extraction analysis

|  | Cd  Frac 5 | Cr  Frac 5 | Cu  Frac 5 | Ni  Frac 5 | Pb  Frac 5 | Zn  Frac 5 |
| --- | --- | --- | --- | --- | --- | --- |
| Os1_In | 0.0254 | 2.98 | 6.65 | 4.65 | 1.52 | 12.5 |
| Os1_Out | 0.0463 | 6.62 | 24.1 | 4.89 | 0.958 | 16.5 |
| Or1_In | 0.0139 | 5.37 | 2.1 | 2.64 | 1.15 | 15.5 |
| Or1_Out | 0.0088 | 7.64 | 3.15 | 3.8 | 1.39 | 15.7 |
| Or2_In | 0.0065 | 13.2 | 3.27 | 6.4 | 1.25 | 22.8 |
| Or2_Out | 0.0198 | 14.5 | 10.2 | 9.06 | 3.61 | 26.4 |
| Or3_In | 0.0081 | 14.1 | 4.74 | 7.41 | 1.22 | 25.1 |
| Or3_Out | 0.00868 | 10 | 7.65 | 5.74 | 1.03 | 20.4 |
| Or4_In | <0.01 | 14.3 | 6.31 | 8.92 | 2.23 | 26.6 |
| Or4_Out | 0.0103 | 14.3 | 6.18 | 7.36 | 2.64 | 25.1 |
| Or5_In | 0.0086 | 3.71 | 1.39 | 1.76 | 0.9 | 10.7 |
| Or5_Out | 0.0101 | 2.98 | 1.21 | 1.53 | 0.93 | 7.91 |
| Or6_In | 0.0043 | 1.95 | 1.47 | 1.04 | 0.917 | 4.55 |
| Or6_Out | 0.00682 | 3.52 | 1.77 | 1.62 | 1.22 | 7.76 |
| S1_In | 0.0312 | 15.1 | 25.3 | 7.53 | 1.27 | 25.2 |
| S1_Out | 0.0311 | 31.9 | 42.6 | 13 | 2.09 | 41.1 |
| S2_In | 0.0169 | 10.4 | 5.31 | 5.11 | 1.44 | 19.7 |
| S2_Out | 0.0143 | 20.4 | 14.9 | 10.6 | 1.32 | 25.8 |
| S3_In | 0.0189 | 11.9 | 7.37 | 5.33 | 0.647 | 20.3 |
| S3_Out | 0.00973 | 15.3 | 13.1 | 5.78 | 0.716 | 12.7 |
| S4_In | 0.0361 | 27.1 | 23.8 | 11.5 | 1.8 | 60.6 |
| S5_In | 0.0284 | 14.5 | 12.9 | 6.6 | 0.943 | 24.8 |
| S5_Out | 0.0175 | 25.1 | 11.7 | 14.9 | 3.13 | 39.7 |
| S6_In | 0.032 | 13.1 | 10.3 | 5.37 | 1.2 | 20.8 |
| S6_Out | 0.0394 | 22.2 | 30.5 | 10.7 | 1.78 | 45.2 |
| V1_In | 0.0354 | 16.9 | 17 | 7.24 | 1.38 | 14 |
| V1_Out | 0.00511 | 1.95 | 1.7 | 1.05 | 0.461 | 4.04 |
| V2_In | 0.0175 | 3.08 | 4.51 | 1.69 | 0.457 | 11.4 |
| V3_In | 0.0266 | 7.09 | 10.9 | 3.92 | 0.849 | 9.88 |
| V3_Out | 0.0254 | 9.73 | 25.2 | 5.96 | 1.1 | 16.9 |
| V4_In | 0.0312 | 13.3 | 12.5 | 7.87 | 1.94 | 31.5 |
| V4_Out | 0.0319 | 14 | 8.04 | 5.64 | 1.48 | 29.6 |
